# Evaluating DF Relaying Energy-Harvesting Systems with Multiple Antennas and Adaptive Transmission over *α*-*μ* Fading Channels

**DOI:** 10.3390/s26206454

**Published:** 2026-10-12

**Authors:** Arfan Ahmed, Ramesh Kumar, Arsalan Ansari, Atif Jamil

**Affiliations:** 1 Department of Electronic Engineering, Dawood University of Engineering and Technology, Karachi 74800, Pakistan; 2 Department of Computer System Engineering, Dawood University of Engineering and Technology, Karachi 74800, Pakistan; ramesh.kumar@duet.edu.pk; 3 Department of Telecommunication Engineering, Dawood University of Engineering and Technology, Karachi 74800, Pakistan; dr.arsalanansari@duet.edu.pk; 4 Department of Computer Science, Dawood University of Engineering and Technology, Karachi 74800, Pakistan; atif.jamil@duet.edu.pk

**Keywords:** adaptive transmission, bit error rate, channel capacity, decode-and-forward, energy harvesting, outage probability

## Abstract

Energy harvesting (EH) is an emerging technology for enabling sustainable wireless communication in sixth-generation (6G) networks, particularly for energy-constrained network nodes that are difficult to maintain or recharge. In this paper, we propose and analyze an adaptive energy-harvesting decode-and-forward (DF) relaying framework over generalized α-μ fading channels. The proposed framework consists of a multi-antenna source, an energy-constrained multi-antenna relay, and a multi-antenna destination, with direct source-to-destination communication assumed infeasible due to severe channel impairments. To sustain relay operation, the relay harvests energy from received radio-frequency (RF) signals using time-switching (TSR) and power-splitting (PSR) protocols, then forwards the decoded information in half-duplex mode. The generalized α-μ distribution provides a unified statistical characterization of wireless propagation across diverse 6G scenarios and encompasses several well-known fading models as special cases, such as Rayleigh, Nakagami-*m*, Weibull, one-sided Gaussian, and exponential fading. The study considers multiple transmission strategies, including optimal rate adaptation (ORA), optimal power and rate adaptation (OPRA), channel inversion with fixed rate (CIFR), and truncated channel inversion with fixed rate (TIFR). Exact-form expressions for the outage probability, Bit error rate (BER) using the moment-generating-function approach, and channel capacity are derived for the considered adaptive transmission strategies. The diversity order is also characterized to evaluate system performance under different numbers of antenna elements, transmit power levels, energy-harvesting ratios, and fading severities. Numerical results from Monte Carlo simulations demonstrate the performance of the proposed framework under different system configurations, including Amazon Kubernetes Service (AKS). The analytical results closely match the simulation results, validating the derived expressions. Overall, the proposed EH-enabled Decode-and-Forward (DF) relaying framework provides a flexible approach to improving reliability, reducing outage probability and BER, and enhancing channel capacity in energy-constrained 6G wireless networks.

## 1. Introduction

Recent advances in sixth-generation (6G) and Internet of Things (IoT) technologies impose new communication requirements, including ultra-high spectral efficiency, massive connectivity, and energy sustainability under power constraints. Besides enhanced mobile broadband, future sixth-generation networks are anticipated to simultaneously enable densely deployed heterogeneous networks with limited lifetimes and challenging battery replacement. These aspects motivate the research community to design and develop innovative communication techniques that enable energy efficiency. One such promising technique is Energy Harvesting (EH) [1,2].

In particular, EH enables wireless devices to harvest energy from various sources, including solar, thermal, wind, and radio-frequency (RF) [3]. Energy can be harvested from RF signals transmitted by a dedicated power station or wireless nodes, enabling simultaneous wireless information and power transfer [4,5]. This approach can enable wireless networks to operate continuously without monitoring battery levels, which is especially advantageous for low-power devices such as IoT devices, sensors, and wearables.

Dual-hop relaying networks have been proposed to improve coverage, reliability, and/or energy efficiency in wireless systems [6,7]. The most common DF protocol for decode-and-forward relaying is used due to its low complexity and noise-reduction capability, in which the signal is decoded, re-encoded, and forwarded at the relay [8]. To maximize energy efficiency, RF-EH-enabled relays usually employ time-switching relaying (TSR) or power-splitting relaying (PSR) protocols to simultaneously harvest energy and process information. Specifically, TSR employs a time-division manner to alternately harvest energy and process information, while PSR utilizes a power-division manner to split the received signal into two portions for EH and information decoding on the same channel and outage probability (OP) [9,10].

Nevertheless, wireless signal propagation in realistic environments is subject to severe multipath fading, shadowing, and path loss, which degrade the performance of wireless communication systems. To mitigate the adverse effects of multipath fading and enhance channel capacity and link reliability, adaptive transmission techniques, optimal rate adaptation (ORA), optimal power and rate adaptation (OPRA), channel inversion with fixed rate (CIFR), and truncated channel inversion with fixed rate (TIFR) have been widely investigated. These adaptive transmission schemes have different policies to adjust the transmit power and/or rate based on instantaneous channel state information (CSI) exploited at the transmitter [11,12].

Most existing studies on RF energy-harvesting relay systems assume classical fading models, such as Rayleigh or Nakagami-*m*. However, these models fail to accurately capture the complexity of real-world wireless propagation in next-generation networks. The generalized α-μ fading model provides a more flexible and realistic statistical framework that encompasses several well-known fading distributions as special cases, including Rayleigh, Nakagami-*m*, Weibull, and one-sided Gaussian fading. This makes it particularly suitable for modeling 6G heterogeneous communication environments. The Simultaneous Wireless Information and Power Transfer (SWIPT) protocol is also used in the methodology.

The novelty of this paper lies in proposing an adaptive energy-harvesting DF relaying framework over generalized α-μ fading channels. The system consists of a multi-antenna source, an energy-constrained multi-antenna relay, and a multi-antenna destination, with direct source-to-destination communication assumed infeasible due to severe channel conditions. The relay harvests energy from the received RF signal using both TSR and PSR protocols and forwards the decoded information in a half-duplex mode. Adaptive transmission strategies are employed to enhance system robustness and spectral efficiency under varying channel conditions.

The paper presents the following core contributions to the problem considered.

- We propose a dual-hop DF relaying-based energy harvesting (DHDF-EH) framework over generalized α-μ fading channels. The system consists of a multi-antenna source, relay, and destination, where the relay operates under energy constraints and harvests energy using both TSR and PSR protocols.
- We incorporate multiple adaptive transmission schemes, including ORA, OPRA, CIFR, and TIFR, to enhance channel capacity under varying rates and powers.
- We derive analytical expressions for key performance metrics, including outage probability, BER using the moment-generating function (MGF) approach, and channel capacity, for the adaptive transmission techniques considered.
- We present an extensive performance evaluation to investigate the impact of key system parameters, including antenna configuration, transmit power, energy-harvesting factors, and fading severity, on overall system performance.
- We simulate the experiments on AKS to validate the performance of the proposed scheme. Monte Carlo simulations are conducted to validate the accuracy of the derived analytical expressions, which show excellent agreement with theoretical results and confirm the effectiveness of the proposed framework for next-generation 6G energy-constrained networks.

The paper includes sections on related work, system model, methodology, evaluation, and conclusion.

## 2. Related Work

EH and cooperative relaying have been emerging research topics for future wireless networks, where energy efficiency and spectrum utilization are important concerns. Some recent work has focused on the simultaneous operation of backscatter communication and EH to enhance the performance and save power of wireless systems. Tran et al. [1] studied the outage probability of a hybrid relay-backscatter system under energy-harvesting constraints, which is applicable to IoT-based 6G systems.

The paper [2] provides an overview of radio-frequency (RF) and EH techniques for wireless, self-powered, battery-less sensors and industrial applications. Advanced rectifier designs for RF energy-harvesting systems operating across multi-band frequencies are discussed in [3]. The interference-assisted and power-splitting-based EH approaches for cooperative relay networks have attracted considerable research attention and have been widely investigated in [4,5], as they can significantly improve harvested energy and system throughput Moreover, multihop and SWIPT-based DF systems have been studied in [12,13], where cooperative communications have been shown to benefit energy-harvesting Internet of Things.

The literature on wireless information and energy transfer in DF relaying systems has grown significantly over the past few years. In particular, the throughput and ergodic capacity of energy-harvesting DF relaying systems have been widely investigated in [14].

Performance analysis of energy-harvesting DF systems under various fading environments and with adaptive transmission policies has been widely studied in the literature. In particular, adaptive power control and capacity analysis of energy-harvesting DF systems under generalized fading channels, including κ-μ, α-μ, and shadowed models, have been widely studied [15,16]. It has been found that channel-adaptive transmission techniques can yield remarkable gains in system throughput and reductions in outage probability. In particular, it has been shown that nonlinear energy harvesting and transceiver impairments significantly affect the outage probability and throughput of SWIPT-based systems, especially at high signal-to-noise ratios (SNRs). On the other hand, cooperative systems employing selective DF relaying protocols and SWIPT under the effects of channel aging have been studied in Furthermore, EH and DF systems with nonlinear EH circuits and hardware impairments have been studied in [17,18,19,20,21,22,23,24,25,26,27].

In addition, recent studies on advanced communication networks with cognitive radio, non-orthogonal multiple access (NOMA), and interference-limited amplify-and-forward (AF) relaying systems with EH have been conducted [28,29,30]. These studies [31,32] suggested the adaptive transmission with the Dual-hop cooperative relaying with beamforming under adaptive transmission in κ-μ shadowed fading and optimize Energy harvesting in α-μ environment under different relaying protocols. This work [33] investigated the outage performance and diversity order of Reconfigurable intelligent surfaces-assisted energy-harvesting (RIS-AEH) DF relaying systems over generalized fading channels. This work [34] presented a unified outage probability framework for dual-hop DF relaying systems with EH over fading channels.

Overall, most of the prior work focuses either on RIS-assisted relaying, unified DF outage analysis, or SWIPT-based selective protocols. Meanwhile, more sophisticated hybrid EH protocols and generalized fading models are rarely investigated. Although considerable effort has been devoted to this area, modeling adaptive transmission in fading channels with energy-harvesting constraints remains challenging, motivating us to study decode-and-forward relaying systems over generalized α-μ fading channels with adaptive transmission schemes.

## 3. System Model and Channel Model

We have used CSI to monitor the sender-to-receiver channel information in the system. This is already deployed and available in this study [35].

### 3.1. System Model

**Figure 1:**
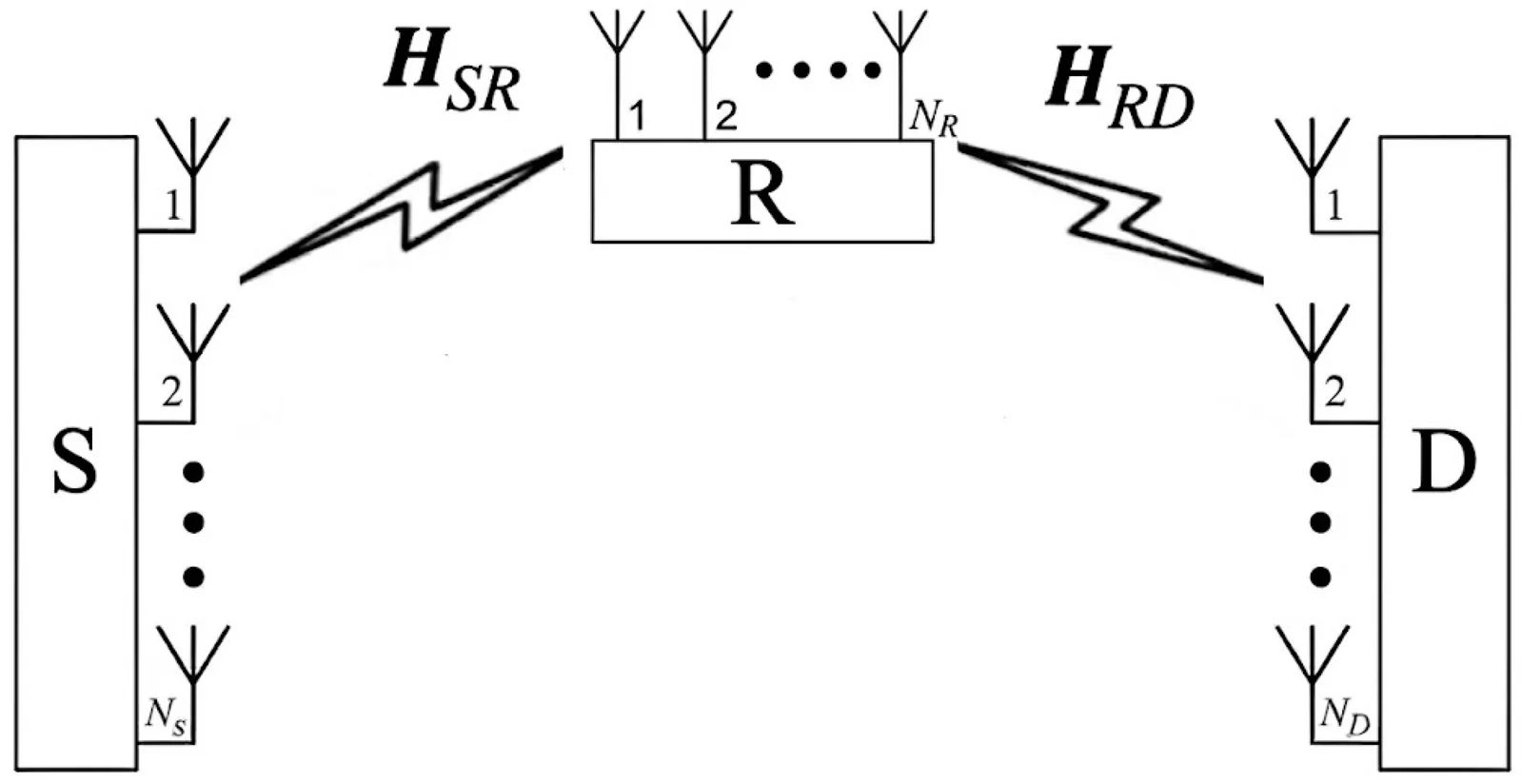
Dual-hop decode-and-forward relaying-based EH system.

**Figure 2:**
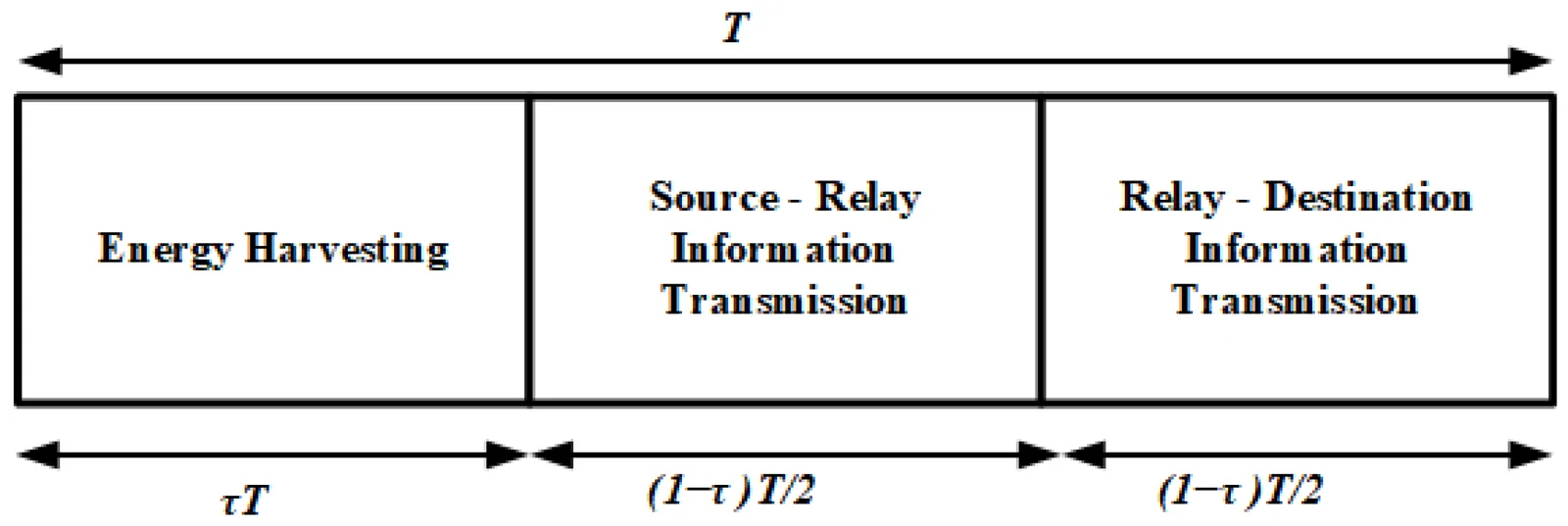
Time Block Mechanism in Time-Switching Relaying Protocol.

We propose a dual-hop DF-based EH system that employs adaptive transmission. The system uses a DF relay with two main approaches: time-switching and power-splitting. In time-switching, the relay collects energy and receives information during separate time intervals. In power-splitting, the relay splits the incoming signal, using one part for energy and the other for forwarding information. The system comprises a source node (S) equipped with $N_{S}$ antennas, a relay (R) with $N_{R}$ antennas and limited energy resources, and a destination (D) node with $N_{D}$ antennas, as illustrated in Figure 1. When severe fading impairs the direct link between the source and destination, the relay maintains communication continuity. The system functions in half-duplex mode, which precludes simultaneous transmission and reception. The relay manages this operational process. The wireless links from the source to the relay and from the relay to the destination are modeled using the α-μ fading distribution, which captures various types of signal variations. At the relay node, the relay harvests energy from the RF source signal and distributes it through both TSR and PSR schemes to help enable communication. The relay’s circuitry needs power to process data, but this power consumption is not considered in the analysis. In TSR, protocol transitions occur as follows: the relay harvests energy and decodes information in separate intervals. The transmission time frame *T* is divided into three distinct periods, as illustrated in Figure 2. During the first period, lasting τT, the relay harvests energy, where τ denotes the time-switching factor, with 0≤τ≤1. In interval I, the S transmits data to the R, while in interval II, the R forwards the decoded data to the D. This configuration ensures equal transmission time in both intervals. At the destination, all incoming signals are combined through maximum-ratio combining (MRC).

We assume that a direct link from the source to the destination is unavailable due to deep fading. Therefore, we are not considering it in Figure 1.

The signal sent from the source and received by the relay node is given as follows.

(1) $$y_{R}=\sqrt{\frac{P_{S}}{d_{SR}^{\;\xi }}}\;H_{SR}\;x_{S}+n_{R}.$$

Here, $x_{S}$ denotes the information signal from the source, $P_{S}$ is the source’s transmit power, $H_{SR}$ is the $N_{S}\times N_{R}$ channel matrix from S to R, ξ denotes the path loss exponent, $d_{SR}$ represents the distance from S to R, and $n_{R}$ is the additive white Gaussian noise (AWGN) at R with variance $\sigma_{R}^{2}$. Using the received signal, the harvested energy at R is described below.

(2) $$E_{H}=\epsilon \;\tau \;P_{S}\;T\;\frac{{∥H_{SR}∥}^{2}}{d_{SR}^{\;\xi }},$$

Here, 0 ≤ ϵ ≤ 1 represents the energy-harvesting efficiency, which primarily depends on the relay circuitry. Having established this, we next clarify the intervals relevant to signal reception at the destination node.

(3) $$y_{D}=\sqrt{\frac{P_{R}}{d_{RD}^{\;\xi }}}\;H_{RD}\;x_{R}+n_{D}.$$

Here, $x_{R}$ stands for the relay signal, $H_{RD}$ is the $N_{R}\times N_{D}$ channel matrix from R to D, and $n_{D}$ represents the AWGN at the D node and has a variance of $\sigma_{D}^{2}$. This variable $P_{R}$ denotes the relay’s transmit power, which the EH sets, as explained below.

(4) $$\begin{array}{cc} P_{R} & =\frac{E_{H}}{\left(1-\tau \right)\left(\frac{T}{2}\right)}\\ \; & =\frac{2\tau \epsilon P_{S}{∥H_{SR}∥}^{2}}{\left(1-\tau \right)d_{SR}^{\;\xi }}. \end{array}$$

The destination signal is calculated by substituting (4) into (3).

(5) $$y_{D}=\sqrt{\frac{2\epsilon \tau P_{S}}{\left(1-\tau \right)\;d_{SR}^{\;\xi }\;d_{RD}^{\;\xi }}}\;H_{SR}\;H_{RD}\;x_{R}+n_{D}.$$

Based on (1) and (5), the SNR at the R and D nodes are as follows

(6) $$\gamma_{R}=\frac{P_{S}\;{∥H_{SR}∥}^{2}}{d_{SR}^{\;\xi }\;\sigma_{R}^{2}},$$

and

(7) $$\gamma_{D}=\frac{2\;\epsilon \;\tau \;P_{S}{∥H_{SR}∥}^{2}{∥H_{RD}∥}^{2}}{\left(1-\tau \right)\;d_{SR}^{\;\xi }\;d_{RD}^{\;\xi }\;\sigma_{D}^{2}}.$$

**Figure 3:**
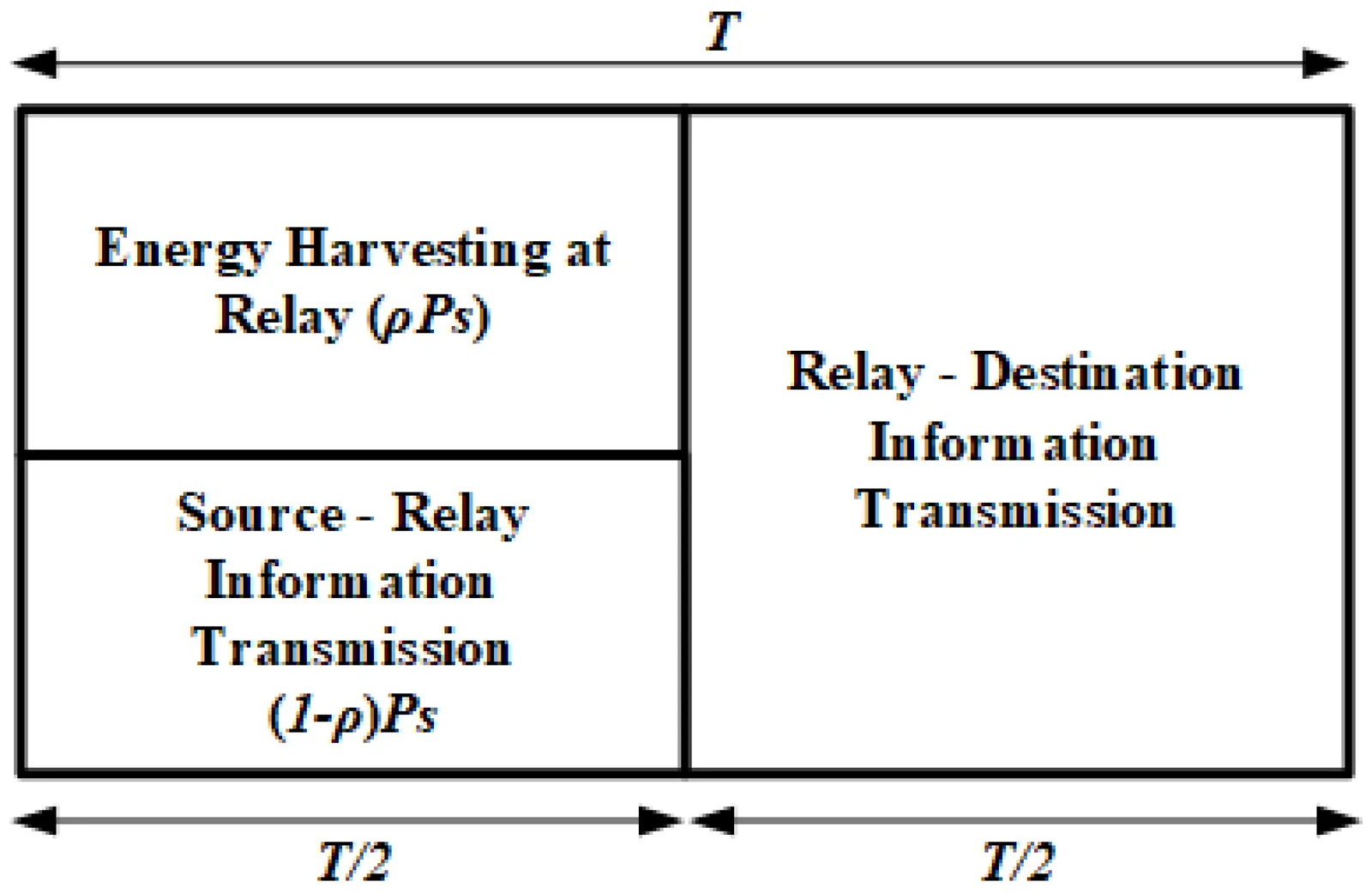
Time block mechanism for power splitting relaying Protocol.

The second scheme, PSR, uses a power splitter to divide the received signal into two parts: $\rho P_{S}$ and $\left(1-\rho \right)P_{S}$, where ρ is the power-splitting ratio, 0≤ρ≤1. Figure 3 illustrates the transmission block at relay node R for energy harvesting and information processing. The block time *T* is divided equally into two slots. In the first slot, the source transmits both information and energy to the relay node. The portion $\rho P_{S}$ is allocated to energy harvesting, while $\left(1-\rho \right)P_{S}$ is allocated to information processing. In the second slot, R uses the harvested energy to forward the decoded signal to D. At D, the received signals are combined using MRC.

The signal received from the S at R is expressed as

(8) $$\sqrt{\rho }\;y_{R}=\sqrt{\frac{\rho P_{S}}{d_{SR}^{\;\xi }}}\;H_{SR}\;x_{S}+\sqrt{\rho }\;n_{A,R}.$$

At the relay, the power splitter first divides $y_{R}$ into two portions. The energy harvester receiver receives the initial portion $\sqrt{\rho }\;y_{R}$ to harvest energy. Next, the information receiver uses the remaining portion, $\sqrt{1-\rho }\;y_{R}$, for information processing. During T/2, the EH receiver accumulates energy from its assigned portion. The relay’s harvested energy is determined as follows.

(9) $$E_{H}=\epsilon \;\rho \;P_{S}\;T\;\frac{{∥H_{SR}∥}^{2}}{2\;d_{SR}^{\;\xi }}.$$

The relay transmitting power $P_{R}$ is obtained as

(10) $$P_{R}=\frac{E_{H}}{T/2}=\epsilon \;\rho \;P_{S}\;\;\frac{∥H_{SR}{∥}^{2}}{d_{SR}^{\;\xi }}.$$

The residual signal observed at the information receiver is presented below

(11) $$\begin{array}{cc} \sqrt{1-\rho }\;y_{R} & =\sqrt{\frac{\left(1-\rho \right)P_{S}}{d_{SR}^{\;\xi }}}\;H_{SR}\;x_{S}+\sqrt{1-\rho }\;n_{A,R}\\ \; & \;+\;n_{C,R}, \end{array}$$

Here, $n_{C,R}$ represents the noise encountered by the information receiver; its variance is specifically denoted as $\sigma_{C,R}^{2}$. We now proceed to the final phase of the process. In the final phase of the communication process, after the initial signal transmission and EH, the DF relay first decodes the signal given by (11), then remodulates it, and finally retransmits it using the harvested energy shown in (9). As a result, the destination node receives the following signal.

(12) $$y_{D}=\sqrt{\frac{P_{R}}{d_{RD}^{\;\xi }}}\;H_{RD}\;x_{R}+n_{D}.$$

Now, substituting (10) into (12), the expression becomes

(13) $$y_{D}=\sqrt{\frac{\epsilon \;\rho \;P_{S}}{d_{SR}^{\;\xi }\;d_{RD}^{\;\xi }}}\;H_{SR}\;H_{RD}\;x_{R}+n_{D}.$$

Equations (11) and (13) provide the SNRs at the R and D nodes. The corresponding SNR at R is

(14) $$\gamma_{R}=\frac{\left(1-\rho \right)P_{s}{\left|H_{SR}\right|}^{2}}{d_{SR}^{\xi }\left[\left(1-\rho \right)\sigma_{A,R}^{2}+\sigma_{C,R}^{2}\right]},$$

and

(15) $$\gamma_{D}=\frac{\epsilon \rho P_{S}{\left|H_{SR}\right|}^{2}{\left|H_{RD}\right|}^{2}}{d_{SR}^{\xi }d_{RD}^{\xi }\sigma_{D}^{2}}.$$

### 3.2. Channel Model

The propagation environment follows the α-μ fading distribution, which models a signal composed of multiple clusters of scattered components propagating through an irregular, nonuniform environment. Within each cluster, the scattered wave components possess random phases but share nearly identical delay characteristics, whereas the delay spread between different clusters is comparatively large. It is assumed that the scattered waves within these clusters have equal power. The envelope of the received signal is calculated by applying a nonlinear transformation to the magnitude of the combined multipath signals. This nonlinearity is expressed as a power parameter that represents the overall signal strength, not merely the resultant magnitude obtained by summing the multipath signals, but rather the magnitude raised to a specific exponent. It is a flexible fading model that encompasses several small-scale fading distributions, including the Rayleigh, Nakagami-m, one-sided Gaussian, negative exponential, and Weibull distributions. Specific values of α and μ correspond to these classical models, as shown in Table 1.

Next, we present the statistics of the channel’s instantaneous SNR, γ, under α-μ fading. The probability density function (PDF) of γ, denoted as $f_{\gamma }\left(\gamma \right)$, describes the distribution of the random variable γ for multiple-antenna cases, as explained in [30].

(16) $$f_{\gamma }\left(\gamma \right)=\frac{\alpha {\left(N\mu \right)}^{N\mu }}{2\;\Gamma \left(N\mu \right)\;{\left(N\bar{\gamma }\right)}^{\frac{\alpha N\mu }{2}}}\;\gamma^{\frac{\alpha N\mu }{2}-1}\;e^{-\;N\mu {\left(\frac{\gamma }{N\bar{\gamma }}\right)}^{\frac{\alpha }{2}}},$$

where the fading nonlinearity parameter α>0 and μ>0 define the inverse normalized variance. Γ(·) represents the Gamma function. $\bar{\gamma }=E\left\{\gamma \right\}$ signifies the average SNR.

## 4. Performance Analysis

In this section, we derive exact analytical expressions for the OP, the channel capacity based on adaptive transmission techniques, and the BER using the moment-generating function.

### 4.1. Outage Probability Analysis

Outage probability refers to the likelihood that the received SNR falls below the specified threshold. The equation of OP is given in [31].

(17) $$\begin{array}{cccc} P_{\mathrm{out}} & =F_{\gamma_{\mathrm{tot}}}\left(\gamma \right)\;\; & =1-\mathrm{Pr}\left[\gamma_{R}>\gamma \right]\mathrm{Pr}\left[\gamma_{D}>\gamma \right] & =1-\int_{\gamma }^{\infty }f_{\gamma_{R}}\left(\gamma_{R}\right)d\gamma_{R}\int_{\gamma }^{\infty }f_{\gamma_{D}}\left(\gamma_{D}\right)d\gamma_{D}, \end{array}$$

where $f_{\gamma_{R}}\left(\gamma \right)$ and $f_{\gamma_{D}}\left(\gamma \right)$ are PDFs for the S to R and R to D hops, are given in (16). $F_{\gamma_{\mathrm{tot}}}\left(\gamma \right)$ denotes the cumulative distribution function of $\gamma_{\mathrm{tot}}$. The symbol Pr[·] represents probability. By substituting (16) into (17) and evaluating the integrals sequentially, we obtain

(18) $$\int_{\gamma }^{\infty }f_{\gamma_{R}}\left(\gamma_{R}\right)d\gamma_{R}=\int_{\gamma }^{\infty }\frac{\alpha {\left(N_{S}\mu \right)}^{N_{S}\mu }\gamma_{R}^{\frac{\alpha N_{S}\mu }{2}-1}}{2{\left(N_{S}{\bar{\gamma }}_{R}\right)}^{\frac{\alpha N_{S}\mu }{2}}\Gamma \left(N_{S}\mu \right)}e^{-N_{S}\mu {\left(\frac{\gamma_{R}}{N_{S}{\bar{\gamma }}_{R}}\right)}^{\frac{\alpha }{2}}}d\gamma_{R},$$

and

(19) $$\int_{\gamma }^{\infty }f_{\gamma_{D}}\left(\gamma_{D}\right)d\gamma_{D}\;=\int_{\gamma }^{\infty }\frac{\alpha {\left(N_{R}\mu \right)}^{N_{R}\mu }\gamma_{D}^{\frac{\alpha N_{R}\mu }{2}-1}}{2{\left(N_{R}{\bar{\gamma }}_{D}\right)}^{\frac{\alpha N_{R}\mu }{2}}\Gamma \left(N_{R}\mu \right)}e^{-N_{R}\mu {\left(\frac{\gamma_{D}}{N_{R}{\bar{\gamma }}_{D}}\right)}^{\frac{\alpha }{2}}}d\gamma_{D}.$$

Next, we solve (18) and (19) by using formula (3.381.9) from [36]. This changes the equation to

(20) $$\int_{\gamma }^{\infty }f_{\gamma_{R}}\left(\gamma_{R}\right)d\gamma_{R}=\frac{\Gamma \left(N_{S}\mu ,N_{S}\mu {\left(\frac{\gamma }{N_{S}{\bar{\gamma }}_{R}}\right)}^{\alpha /2}\right)}{\Gamma (N_{S}\mu )}.$$

(21) $$\int_{\gamma }^{\infty }f_{\gamma_{D}}\left(\gamma_{D}\right)d\gamma_{D}\;=\frac{\Gamma \left(N_{R}\mu ,N_{R}\mu {\left(\frac{\gamma }{N_{R}{\bar{\gamma }}_{D}}\right)}^{\alpha /2}\right)}{\Gamma (N_{R}\mu )}.$$

The expression for OP is obtained as follows by substituting (20) and (21) into (17).

(22) $$\begin{array}{ccc} P_{\mathrm{out}} & =F_{\gamma_{\mathrm{tot}}}\left(\gamma \right)\;\; & =1-\left(\frac{\Gamma \left(N_{S}\mu ,N_{S}\mu {\left(\frac{\gamma }{N_{S}{\bar{\gamma }}_{R}}\right)}^{\alpha /2}\right)}{\Gamma (N_{S}\mu )}\right)\left(\frac{\Gamma \left(N_{R}\mu ,N_{R}\mu {\left(\frac{\gamma }{N_{R}{\bar{\gamma }}_{D}}\right)}^{\alpha /2}\right)}{\Gamma (N_{R}\mu )}\right). \end{array}$$

This is the final outage probability expression for both TSR and PSR protocols. For TSR, the threshold is $\gamma =2^{\frac{2r}{1-\tau }}-1$. For PSR, the threshold is $\gamma =2^{2r}-1$, here Γ(,) denotes upper incomplete gamma function, and r is the target transmission rate [32]. The next step is to derive the corresponding PDF expression.

(23) $$\begin{array}{cc} f_{\gamma_{\mathrm{tot}}}\left(\gamma \right) & =\frac{d}{d\gamma }\{1-\left(\frac{\Gamma \left(N_{S}\mu ,\;N_{S}\mu {\left(\frac{\gamma }{N_{S}{\bar{\gamma }}_{R}}\right)}^{\alpha /2}\right)}{\Gamma (N_{S}\mu )}\right)\\ \; & \;\times \left(\frac{\Gamma \left(N_{R}\mu ,\;N_{R}\mu {\left(\frac{\gamma }{N_{R}{\bar{\gamma }}_{D}}\right)}^{\alpha /2}\right)}{\Gamma (N_{R}\mu )}\right)\}. \end{array}$$

Solving (23) by the product rule of derivatives and then applying identities (8.356.4), (8.352.4), and (8.339.1) from [36], leading to the final result as given below

(24) $$\begin{array}{cc} f_{\gamma_{\mathrm{tot}}}\left(\gamma \right) & =\frac{\alpha }{2}\Delta_{R}\;\gamma^{\frac{\alpha N_{S}\mu }{2}-1}e^{-\Omega \gamma^{\frac{\alpha }{2}}}\sum_{i=0}^{N_{S}\mu -1}\frac{{\left(\Omega_{D}\gamma^{\frac{\alpha }{2}}\right)}^{i}}{i!}\\ \; & \;+\;\frac{\alpha }{2}\Delta_{D}\;\gamma^{\frac{\alpha N_{R}\mu }{2}-1}e^{-\Omega \gamma^{\frac{\alpha }{2}}}\sum_{j=0}^{N_{R}\mu -1}\frac{{\left(\Omega_{R}\gamma^{\frac{\alpha }{2}}\right)}^{j}}{j!}. \end{array}$$

Here,

$$\begin{array}{cc} \Delta_{R} & =\frac{{\left(N_{S}\mu \right)}^{N_{S}\mu }}{\Gamma (N_{S}\mu )}{\left(N_{S}{\bar{\gamma }}_{R}\right)}^{-\frac{\alpha N_{S}\mu }{2}},\\ \Delta_{D} & =\frac{{\left(N_{R}\mu \right)}^{N_{R}\mu }}{\Gamma (N_{R}\mu )}{\left(N_{R}{\bar{\gamma }}_{D}\right)}^{-\frac{\alpha N_{R}\mu }{2}},\\ \Omega_{R} & =N_{S}\mu {\left(N_{S}{\bar{\gamma }}_{R}\right)}^{-\frac{\alpha }{2}},\\ \Omega_{D} & =N_{R}\mu {\left(N_{R}{\bar{\gamma }}_{D}\right)}^{-\frac{\alpha }{2}},\\ \Omega & =\Omega_{R}+\Omega_{D}. \end{array}$$

### 4.2. Channel Capacity Analysis

Channel capacity is determined by averaging Shannon’s AWGN channel capacity over the probability distribution of the small-scale fading model, which in this case is the α-μ distribution. The formula $C=E\left[\log_{2}\left(1+\gamma \right)\right]$ is applied, where *C* represents the normalized channel capacity in bits per second per hertz. With this SNR framework in place, we next analyze and compute the channel capacity for several adaptive transmission strategies: ORA, OPRA, CIFR, and TIFR.

#### 4.2.1. Optimal Rate Adaptation

In the ORA scheme, we determine channel capacity and optimize rate adaptation at constant transmit power using the following process [37]

(25) $$C_{\mathrm{ORA}}=B\int_{0}^{\infty }\log_{2}\left(1+\gamma \right)\;f_{\gamma_{\mathrm{tot}}}\left(\gamma \right)\;d\gamma ,$$

Here, *B* denotes the channel bandwidth. We can also write (25) in this form

(26) $$C_{\mathrm{ORA}}=\frac{B}{\ln2}\int_{0}^{\infty }\ln\left(1+\gamma \right)\;f_{\gamma_{\mathrm{tot}}}\left(\gamma \right)\;d\gamma .$$

We use integration by parts to evaluate the capacity integral in (26) as follows

$$u=\ln\left(1+\gamma \right),\;dv=f_{\gamma_{\mathrm{tot}}}\left(\gamma \right)\;d\gamma .$$

In this derivation, we find the term *v* as $v=1-F_{\gamma_{\mathrm{tot}}}\left(\gamma \right)$, instead of $F_{\gamma_{\mathrm{tot}}}\left(\gamma \right)$. This is because the original integral is defined on [0,∞]. Once we make these substitutions and perform integration by parts, we can rewrite the capacity integral in (26) as shown below

(27) $$C_{\mathrm{ORA}}=\frac{B}{\ln2}\int_{0}^{\infty }\frac{1-F_{\gamma_{\mathrm{tot}}}\left(\gamma \right)}{1+\gamma }\;d\gamma .$$

Substituting (22) into (27) and solving the expression with the help of (8.356.3), (8.352.4), and (8.339.1) as in [36], the obtained expression is

(28) $$\begin{array}{cc} C_{\mathrm{ORA}} & =\frac{B}{\ln2}\sum_{i=0}^{N_{S}\mu -1}\sum_{j=0}^{N_{R}\mu -1}\frac{\Omega_{R}^{i}\Omega_{D}^{j}}{i!\;j!}\\ \; & \;\times \int_{0}^{\infty }\frac{\gamma^{\frac{\alpha }{2}\left(i+j\right)}e^{-\Omega \gamma^{\frac{\alpha }{2}}}}{1+\gamma }\;d\gamma . \end{array}$$

Now, solving the above integral with the help of (1.112.1) and (3.326.2) as in [36], after simplification, we obtained the channel capacity expression

(29) $$\begin{array}{cc} C_{\mathrm{ORA}} & =\frac{\alpha B}{2\ln2}\sum_{i=0}^{N_{S}\mu -1}\sum_{j=0}^{N_{R}\mu -1}\frac{\Omega_{R}^{i}\Omega_{D}^{j}}{i!\;j!}\\ \; & \;\times \sum_{q=0}^{\infty }\frac{{\left(-1\right)}^{q}}{\Omega^{i+j+\frac{2(q+1)}{\alpha }}}\Gamma \;\left(i+j+\frac{2(q+1)}{\alpha }\right). \end{array}$$

#### 4.2.2. Optimal Power and Rate Adaptation

In the OPRA scheme, each source adjusts its transmission power and data rate to changing channel conditions. Channel capacity for this method is provided in [37]

(30) $$C_{\mathrm{OPRA}}=B\int_{\gamma_{0}}^{\infty }\log_{2}\;\left(\frac{\gamma }{\gamma_{0}}\right)f_{\gamma_{\mathrm{tot}}}\left(\gamma \right)\;d\gamma .$$

We can also write (30) in this form

(31) $$C_{\mathrm{OPRA}}=\frac{B}{\ln2}\int_{\gamma_{0}}^{\infty }\ln\;\left(\frac{\gamma }{\gamma_{0}}\right)f_{\gamma_{\mathrm{tot}}}\left(\gamma \right)\;d\gamma .$$

If the instantaneous SNR falls below $\gamma_{0}$, communication between the S and D nodes stops. Therefore, the following condition must be satisfied, as shown in [38]

(32) $$\int_{\gamma_{0}}^{\infty }\frac{\gamma -\gamma_{0}}{\gamma \;\gamma_{0}}f_{\gamma_{\mathrm{tot}}}\left(\gamma \right)\;d\gamma =1.$$

The OPRA scheme requires both S and D nodes to have perfect channel state information, enabling accurate capacity determination [39]. S adjusts its transmission rate and power in real time, using higher values when the channel is good and lower values when the channel is poor.

The capacity integral in (31) can be evaluated using integration by parts. Select

$$u=\ln\;\left(\frac{\gamma }{\gamma_{0}}\right),\;dv=f_{\gamma_{\mathrm{tot}}}\left(\gamma \right)\;d\gamma .$$

In this derivation, note that the term *v* is given by $v=1-F_{\gamma_{\mathrm{tot}}}\left(\gamma \right)$, instead of $F_{\gamma_{\mathrm{tot}}}\left(\gamma \right)$, because the original integral is taken over the interval $\left[\gamma_{0},\infty \right]$. After these substitutions and integration by parts, the capacity integral in (31) is rewritten as

(33) $$C_{\mathrm{OPRA}}=\frac{B}{\ln2}\int_{\gamma_{0}}^{\infty }\frac{1-F_{\gamma_{\mathrm{tot}}}\left(\gamma \right)}{\gamma }\;d\gamma .$$

Next, substitute (22) into (33). Simplify the expression using (8.356.3), (8.352.4), and (8.339.1) as shown in [36] to solve the expression

(34) $$\begin{array}{cc} C_{\mathrm{OPRA}} & =\frac{B}{\ln2}\sum_{i=0}^{N_{S}\mu -1}\sum_{j=0}^{N_{R}\mu -1}\frac{\Omega_{R}^{i}\Omega_{D}^{j}}{i!\;j!}\\ \; & \;\times \int_{\gamma_{0}}^{\infty }\gamma^{\frac{\alpha }{2}\left(i+j\right)-1}e^{-\Omega \gamma^{\frac{\alpha }{2}}}\;d\gamma . \end{array}$$

Using the identity given in (3.381.9) of [36], the above integral is evaluated, and after simplification, the following expression is obtained.

(35) $$\begin{array}{cc} C_{\mathrm{OPRA}} & =\frac{2B}{\alpha \ln2}\sum_{i=0}^{N_{S}\mu -1}\sum_{j=0}^{N_{R}\mu -1}\frac{\Omega_{R}^{i}\Omega_{D}^{j}}{i!\;j!}\\ \; & \;\times \Omega^{-(i+j)}\Gamma \;\left(i+j,\;\Omega \gamma_{0}^{\frac{\alpha }{2}}\right). \end{array}$$

#### 4.2.3. Channel Inversion with Fixed Rate

Under this transmission policy, the transmitter adjusts its power to maintain a constant SNR at the receiver. This method requires complete knowledge of channel envelope variations. Consequently, both modulation and code rate remain fixed. After channel inversion, the receiver experiences a time-invariant channel. For this policy, the channel capacity is defined as [37].

(36) $$C_{\mathrm{CIFR}}=B\log_{2}\;\left(1+{\left[\int_{0}^{\infty }\frac{1}{\gamma }f_{\gamma_{\mathrm{tot}}}\left(\gamma \right)\;d\gamma \right]}^{-1}\right).$$

The expression for $C_{\mathrm{CIFR}}$ does not provide a practical representation of the channel capacity under the CIFR assumption, as it requires excessively high transmit power to counteract deep fading, particularly in the low-SNR regime. Now, putting (24) into (36), we have

(37) $$\begin{array}{cc} C_{\mathrm{CIFR}} & =B\log_{2}\;(1+[\frac{\alpha }{2}\Delta_{R}\sum_{i=0}^{N_{S}\mu -1}\frac{\Omega_{D}^{i}}{i!}\\ \; & \;\times \int_{0}^{\infty }\gamma^{\frac{\alpha }{2}\left(N_{S}\mu +i\right)-2}e^{-\Omega \gamma^{\frac{\alpha }{2}}}\;d\gamma \\ \; & \;+\;\frac{\alpha }{2}\Delta_{D}\sum_{j=0}^{N_{R}\mu -1}\frac{\Omega_{R}^{j}}{j!}\\ \; & \;\times \int_{0}^{\infty }\gamma^{\frac{\alpha }{2}\left(N_{R}\mu +j\right)-2}e^{-\Omega \gamma^{\frac{\alpha }{2}}}\;d\gamma {]}^{-1}). \end{array}$$

Now, we obtain an expression for channel capacity as follows, using (3.326.2) in [36] to evaluate the above integrals.

(38) $$\begin{array}{cc} C_{\mathrm{CIFR}} & =B\log_{2}\;(1+[\Delta_{R}\sum_{i=0}^{N_{S}\mu -1}\frac{\Omega_{D}^{i}}{i!}\\ \; & \;\times \Omega^{-\left(N_{S}\mu +i-\frac{2}{\alpha }\right)}\Gamma \;\left(N_{S}\mu +i-\frac{2}{\alpha }\right)\\ \; & \;+\Delta_{D}\sum_{j=0}^{N_{R}\mu -1}\frac{\Omega_{R}^{j}}{j!}\\ \; & \;\times \Omega^{-\left(N_{R}\mu +j-\frac{2}{\alpha }\right)}\Gamma \;\left(N_{R}\mu +j-\frac{2}{\alpha }\right){]}^{-1}). \end{array}$$

#### 4.2.4. Truncated Channel Inversion with Fixed Rate

Based on the CIFR approach, we introduce truncated channel inversion with fixed rate, applying channel inversion only when the instantaneous SNR exceeds the cutoff value $\gamma_{0}$. Based on this operating principle, the achievable capacity of the system employing the TIFR strategy is obtained using the methodology outlined in [37], yielding the following expression.

(39) $$\begin{array}{cc} C_{\mathrm{TIFR}} & =B\;\log_{2}\left(1+\frac{1}{\int_{\gamma_{0}}^{\infty }\frac{1}{\gamma }\;f_{\gamma_{\mathrm{tot}}}\left(\gamma \right)\;d\gamma }\right)\\ \; & \;\times \left(1-P_{\mathrm{out}}\right). \end{array}$$

Now, inserting (24) into (39), we obtain

(40) $$\begin{array}{cc} C_{\mathrm{TIFR}} & =B\log_{2}(1+[\frac{\alpha }{2}\Delta_{R}\sum_{i=0}^{N_{S}\mu -1}\frac{\Omega_{D}^{i}}{i!}\int_{\gamma_{0}}^{\infty }\gamma^{\frac{\alpha }{2}\left(N_{S}\mu +i\right)-2}e^{-\Omega \gamma^{\frac{\alpha }{2}}}\;d\gamma \\ \; & \;+\;\frac{\alpha }{2}\Delta_{D}\sum_{j=0}^{N_{R}\mu -1}\frac{\Omega_{R}^{j}}{j!}\int_{\gamma_{0}}^{\infty }\gamma^{\frac{\alpha }{2}\left(N_{R}\mu +j\right)-2}e^{-\Omega \gamma^{\frac{\alpha }{2}}}\;d\gamma {]}^{-1})\left(1-P_{\mathrm{out}}\right). \end{array}$$

Solving the above integral with the help of (3.381.9) in [36], we obtained the expression for channel capacity

(41) $$\begin{array}{cc} C_{\mathrm{TIFR}} & =B\;\log_{2}(1+[\Delta_{R}\sum_{i=0}^{N_{S}\mu -1}\frac{\Omega_{D}^{i}}{i!}\Omega^{-(N_{S}\mu +i-\frac{2}{\alpha })}\\ \; & \;\times \Gamma \left(N_{S}\mu +i-\frac{2}{\alpha },\Omega \;\gamma_{0}^{\frac{\alpha }{2}}\right)\\ \; & \;+\Delta_{D}\sum_{j=0}^{N_{R}\mu -1}\frac{\Omega_{R}^{j}}{j!}\Omega^{-(N_{R}\mu +j-\frac{2}{\alpha })}\\ \; & \;\times \Gamma \left(N_{R}\mu +j-\frac{2}{\alpha },\Omega \;\gamma_{0}^{\frac{\alpha }{2}}\right){]}^{-1})\\ \; & \;\times \left(1-P_{\mathrm{out}}\right). \end{array}$$

### 4.3. Bit Error Rate Analysis

In this study, we employ the method of moment generating functions to derive an analytical expression for the BER.

MGF is a key feature of any probability distribution. It offers a convenient way to study statistical properties and is especially important for multipath fading models in wireless infrastructure. The MGF helps evaluate the performance of wireless infrastructure systems. For example, calculating the bit error rate is important in some situations, and this process becomes much easier with a good understanding of the MGF. The MGF for the proposed fading model is provided in [40] as follows:

(42) $$M_{\gamma_{\mathrm{tot}}}\left(w\right)=w\int_{0}^{\infty }e^{-w\gamma }F_{\gamma_{\mathrm{tot}}}\left(\gamma \right)\;d\gamma .$$

Using identity (6.2.3) as in [41], as given below, we substitute it into (22) and obtain

$$Q\left(a,x\right)=\frac{\Gamma (a,x)}{\Gamma (a)}.$$

(43) $$F_{\gamma_{\mathrm{tot}}}\left(\gamma \right)=1-Q\;\left(N_{S}\mu ,\;N_{S}\mu {\left(\frac{\gamma }{N_{S}{\bar{\gamma }}_{R}}\right)}^{\alpha /2}\right)Q\;\left(N_{R}\mu ,\;N_{R}\mu {\left(\frac{\gamma }{N_{R}{\bar{\gamma }}_{D}}\right)}^{\alpha /2}\right).$$

The identities (6.2.1) and (6.2.3) in [41] and (8.354.1) in [36], as given below.

Q(a,x)=1−P(a,x),

$$P\left(a,x\right)=\frac{\gamma (a,x)}{\Gamma (a)},$$

and

$$P\left(a,x\right)=\frac{1}{\Gamma (a)}\sum_{k=0}^{\infty }\frac{\left(a+k\right){\left(-1\right)}^{k}}{k!}x^{a+k}.$$

Now, using the above identities and solving (43), after simplification, we obtained

(44) $$\begin{array}{cc} F_{\gamma_{\mathrm{tot}}}\left(\gamma \right)= & \frac{1}{\Gamma (N_{S}\mu )}\sum_{k=0}^{\infty }\frac{{\left(-1\right)}^{k}}{k!}\left(N_{S}\mu +k\right){\left(\frac{N_{S}\mu }{{\left(N_{S}{\bar{\gamma }}_{R}\right)}^{\alpha /2}}\right)}^{N_{S}\mu +k}\gamma^{\frac{\alpha }{2}\left(N_{S}\mu +k\right)}\\ \; & +\frac{1}{\Gamma (N_{R}\mu )}\sum_{\ell =0}^{\infty }\frac{{\left(-1\right)}^{\ell }}{\ell !}\left(N_{R}\mu +\ell \right){\left(\frac{N_{R}\mu }{{\left(N_{R}{\bar{\gamma }}_{D}\right)}^{\alpha /2}}\right)}^{N_{R}\mu +\ell }\gamma^{\frac{\alpha }{2}\left(N_{R}\mu +\ell \right)}\\ \; & -\left[\frac{1}{\Gamma (N_{S}\mu )}\sum_{k=0}^{\infty }\frac{{\left(-1\right)}^{k}}{k!}\left(N_{S}\mu +k\right){\left(\frac{N_{S}\mu }{{\left(N_{S}{\bar{\gamma }}_{R}\right)}^{\alpha /2}}\right)}^{N_{S}\mu +k}\gamma^{\frac{\alpha }{2}\left(N_{S}\mu +k\right)}\right]\\ \; & \;\times \left[\frac{1}{\Gamma (N_{R}\mu )}\sum_{\ell =0}^{\infty }\frac{{\left(-1\right)}^{\ell }}{\ell !}\left(N_{R}\mu +\ell \right){\left(\frac{N_{R}\mu }{{\left(N_{R}{\bar{\gamma }}_{D}\right)}^{\alpha /2}}\right)}^{N_{R}\mu +\ell }\gamma^{\frac{\alpha }{2}\left(N_{R}\mu +\ell \right)}\right]. \end{array}$$

Now substitute (44) into (42) and solve the integral.

(45) $$\begin{array}{cc} M_{\gamma_{\mathrm{tot}}}\left(w\right)= & w\int_{0}^{\infty }e^{-w\gamma }[\frac{1}{\Gamma (N_{S}\mu )}\sum_{k=0}^{\infty }\frac{{\left(-1\right)}^{k}}{k!}\left(N_{S}\mu +k\right){\left(\frac{N_{S}\mu }{{\left(N_{S}{\bar{\gamma }}_{R}\right)}^{\alpha /2}}\right)}^{N_{S}\mu +k}\gamma^{\frac{\alpha }{2}\left(N_{S}\mu +k\right)}\\ \; & +\frac{1}{\Gamma (N_{R}\mu )}\sum_{\ell =0}^{\infty }\frac{{\left(-1\right)}^{\ell }}{\ell !}\left(N_{R}\mu +\ell \right){\left(\frac{N_{R}\mu }{{\left(N_{R}{\bar{\gamma }}_{D}\right)}^{\alpha /2}}\right)}^{N_{R}\mu +\ell }\gamma^{\frac{\alpha }{2}\left(N_{R}\mu +\ell \right)}\\ \; & -\left(\frac{1}{\Gamma (N_{S}\mu )}\sum_{k=0}^{\infty }\frac{{\left(-1\right)}^{k}}{k!}\left(N_{S}\mu +k\right){\left(\frac{N_{S}\mu }{{\left(N_{S}{\bar{\gamma }}_{R}\right)}^{\alpha /2}}\right)}^{N_{S}\mu +k}\gamma^{\frac{\alpha }{2}\left(N_{S}\mu +k\right)}\right)\\ \; & \;\times \left(\frac{1}{\Gamma (N_{R}\mu )}\sum_{\ell =0}^{\infty }\frac{{\left(-1\right)}^{\ell }}{\ell !}\left(N_{R}\mu +\ell \right){\left(\frac{N_{R}\mu }{{\left(N_{R}{\bar{\gamma }}_{D}\right)}^{\alpha /2}}\right)}^{N_{R}\mu +\ell }\gamma^{\frac{\alpha }{2}\left(N_{R}\mu +\ell \right)}\right)]\;d\gamma . \end{array}$$

For simplicity, we solve each integral term individually.

(46) $$\begin{array}{cc} I_{1}\left(w\right)= & \frac{w}{\Gamma (N_{S}\mu )}\sum_{k=0}^{\infty }\frac{{\left(-1\right)}^{k}{\left(\frac{N_{S}\mu }{{\left(N_{S}{\bar{\gamma }}_{R}\right)}^{\alpha /2}}\right)}^{N_{S}\mu +k}}{k!(N_{S}\mu +k)}\\ \; & \times \int_{0}^{\infty }\gamma^{\frac{\alpha }{2}\left(N_{S}\mu +k\right)}e^{-w\gamma }\;d\gamma . \end{array}$$

The integral in the above equation is solved by using (3.326.2) in [36].

(47) $$\begin{array}{cc} I_{1}\left(w\right)= & \frac{1}{\Gamma (N_{S}\mu )}\sum_{k=0}^{\infty }\frac{{\left(-1\right)}^{k}{\left(\frac{N_{S}\mu }{{\left(N_{S}{\bar{\gamma }}_{R}\right)}^{\alpha /2}}\right)}^{N_{S}\mu +k}}{k!(N_{S}\mu +k)}\\ \; & \times \;\frac{\Gamma \left(1+\frac{\alpha }{2}\left(N_{S}\mu +k\right)\right)}{w^{\frac{\alpha }{2}\left(N_{S}\mu +k\right)}}. \end{array}$$

Similarly,

(48) $$\begin{array}{cc} I_{2}\left(w\right)= & \frac{1}{\Gamma (N_{R}\mu )}\sum_{\ell =0}^{\infty }\frac{{\left(-1\right)}^{\ell }{\left(\frac{N_{R}\mu }{{\left(N_{R}{\bar{\gamma }}_{D}\right)}^{\alpha /2}}\right)}^{N_{R}\mu +\ell }}{\ell !(N_{R}\mu +\ell )}\\ \; & \times \;\frac{\Gamma \left(1+\frac{\alpha }{2}\left(N_{R}\mu +\ell \right)\right)}{w^{\frac{\alpha }{2}\left(N_{R}\mu +\ell \right)}}. \end{array}$$

(49) $$\begin{array}{cc} I_{12}\left(w\right)= & \frac{w}{\Gamma \left(N_{S}\mu \right)\Gamma \left(N_{R}\mu \right)}\sum_{k=0}^{\infty }\sum_{\ell =0}^{\infty }\frac{{\left(-1\right)}^{k+\ell }}{k!\;\ell !\left(N_{S}\mu +k\right)\left(N_{R}\mu +\ell \right)}\\ \; & \times \;{\left(\frac{N_{S}\mu }{{\left(N_{S}{\bar{\gamma }}_{R}\right)}^{\alpha /2}}\right)}^{N_{S}\mu +k}{\left(\frac{N_{R}\mu }{{\left(N_{R}{\bar{\gamma }}_{D}\right)}^{\alpha /2}}\right)}^{N_{R}\mu +\ell }\\ \; & \times \;\int_{0}^{\infty }\gamma^{\frac{\alpha }{2}\left(N_{S}\mu +N_{R}\mu +k+\ell \right)}e^{-w\gamma }\;d\gamma . \end{array}$$

The integral in the above equation is solved by using (3.326.2) in [36].

(50) $$\begin{array}{cc} I_{12}\left(w\right)= & \frac{1}{\Gamma \left(N_{S}\mu \right)\Gamma \left(N_{R}\mu \right)}\sum_{k=0}^{\infty }\sum_{\ell =0}^{\infty }\frac{{\left(-1\right)}^{k+\ell }}{k!\;\ell !\left(N_{S}\mu +k\right)\left(N_{R}\mu +\ell \right)}\\ \; & \times \;{\left(\frac{N_{S}\mu }{{\left(N_{S}{\bar{\gamma }}_{R}\right)}^{\alpha /2}}\right)}^{N_{S}\mu +k}{\left(\frac{N_{R}\mu }{{\left(N_{R}{\bar{\gamma }}_{D}\right)}^{\alpha /2}}\right)}^{N_{R}\mu +\ell }\\ \; & \times \;\frac{\Gamma \left(1+\frac{\alpha }{2}\left(N_{S}\mu +N_{R}\mu +k+\ell \right)\right)}{w^{\frac{\alpha }{2}\left(N_{S}\mu +N_{R}\mu +k+\ell \right)}}. \end{array}$$

Substituting (47), (48), and (50) into (45) produced the MGF.

(51) $$\begin{array}{cc} M_{\gamma_{\mathrm{tot}}}\left(w\right)= & \frac{1}{\Gamma (N_{S}\mu )}\sum_{k=0}^{\infty }\frac{{\left(-1\right)}^{k}}{k!(N_{S}\mu +k)}{\left(\frac{N_{S}\mu }{{\left(N_{S}{\bar{\gamma }}_{R}\right)}^{\alpha /2}}\right)}^{N_{S}\mu +k}\frac{\Gamma \left(1+\frac{\alpha }{2}\left(N_{S}\mu +k\right)\right)}{w^{\frac{\alpha }{2}\left(N_{S}\mu +k\right)}}\\ \; & +\;\frac{1}{\Gamma (N_{R}\mu )}\sum_{\ell =0}^{\infty }\frac{{\left(-1\right)}^{\ell }}{\ell !(N_{R}\mu +\ell )}{\left(\frac{N_{R}\mu }{{\left(N_{R}{\bar{\gamma }}_{D}\right)}^{\alpha /2}}\right)}^{N_{R}\mu +\ell }\frac{\Gamma \left(1+\frac{\alpha }{2}\left(N_{R}\mu +\ell \right)\right)}{w^{\frac{\alpha }{2}\left(N_{R}\mu +\ell \right)}}\\ \; & -\;\frac{1}{\Gamma \left(N_{S}\mu \right)\Gamma \left(N_{R}\mu \right)}\sum_{k=0}^{\infty }\sum_{\ell =0}^{\infty }\frac{{\left(-1\right)}^{k+\ell }}{k!\;\ell !\left(N_{S}\mu +k\right)\left(N_{R}\mu +\ell \right)}\\ \; & \times \;{\left(\frac{N_{S}\mu }{{\left(N_{S}{\bar{\gamma }}_{R}\right)}^{\alpha /2}}\right)}^{N_{S}\mu +k}{\left(\frac{N_{R}\mu }{{\left(N_{R}{\bar{\gamma }}_{D}\right)}^{\alpha /2}}\right)}^{N_{R}\mu +\ell }\\ \; & \times \;\frac{\Gamma \left(1+\frac{\alpha }{2}\left(N_{S}\mu +N_{R}\mu +k+\ell \right)\right)}{w^{\frac{\alpha }{2}\left(N_{S}\mu +N_{R}\mu +k+\ell \right)}}. \end{array}$$

The BER for a highly coherent modulation scheme is obtained using the moment-generating function approach, as follows [42]. This approach provides a convenient analytical framework for evaluating the BER over the considered fading channels by incorporating the statistical characteristics of the end-to-end instantaneous SNR. Accordingly, the BER is expressed in terms of the MGF of the total instantaneous SNR, which enables the effects of the source-relay and relay-destination links to be systematically incorporated into the overall error-performance analysis. Thus, the BER can be evaluated by integrating the MGF of the total SNR over the modulation-dependent angular parameter.

(52) $${\bar{P}}_{b}=\frac{1}{\pi }\int_{0}^{\pi /2}M_{\gamma_{\mathrm{tot}}}\left(\frac{\psi }{\sin^{2}\theta }\right)d\theta .$$

(53) $$\begin{array}{cc} {\bar{P}}_{b}= & \frac{1}{\pi }\int_{0}^{\pi /2}\frac{\psi }{\sin^{2}\theta }[\frac{1}{\Gamma (N_{S}\mu )}\sum_{k=0}^{\infty }\frac{{\left(-1\right)}^{k}}{k!(N_{S}\mu +k)}{\left(\frac{N_{S}\mu }{{\left(N_{S}{\bar{\gamma }}_{R}\right)}^{\alpha /2}}\right)}^{N_{S}\mu +k}\frac{\Gamma \left(1+\frac{\alpha }{2}\left(N_{S}\mu +k\right)\right)}{w^{\frac{\alpha }{2}\left(N_{S}\mu +k\right)}}\\ \; & +\;\frac{1}{\Gamma (N_{R}\mu )}\sum_{\ell =0}^{\infty }\frac{{\left(-1\right)}^{\ell }}{\ell !(N_{R}\mu +\ell )}{\left(\frac{N_{R}\mu }{{\left(N_{R}{\bar{\gamma }}_{D}\right)}^{\alpha /2}}\right)}^{N_{R}\mu +\ell }\frac{\Gamma \left(1+\frac{\alpha }{2}\left(N_{R}\mu +\ell \right)\right)}{w^{\frac{\alpha }{2}\left(N_{R}\mu +\ell \right)}}\\ \; & -\;\frac{1}{\Gamma \left(N_{S}\mu \right)\Gamma \left(N_{R}\mu \right)}\sum_{k=0}^{\infty }\sum_{\ell =0}^{\infty }\frac{{\left(-1\right)}^{k+\ell }}{k!\ell !\left(N_{S}\mu +k\right)\left(N_{R}\mu +\ell \right)}{\left(\frac{N_{S}\mu }{{\left(N_{S}{\bar{\gamma }}_{R}\right)}^{\alpha /2}}\right)}^{N_{S}\mu +k}\\ \; & \;\times \;{\left(\frac{N_{R}\mu }{{\left(N_{R}{\bar{\gamma }}_{D}\right)}^{\alpha /2}}\right)}^{N_{R}\mu +\ell }\frac{\Gamma \left(1+\frac{\alpha }{2}\left(N_{S}\mu +N_{R}\mu +k+\ell \right)\right)}{w^{\frac{\alpha }{2}\left(N_{S}\mu +N_{R}\mu +k+\ell \right)}}]d\theta . \end{array}$$

Let

$$w=\frac{\psi }{\sin^{2}\theta }.$$

(54) $$w^{-\nu }={\left(\frac{\psi }{\sin^{2}\theta }\right)}^{-\nu }=\frac{\sin^{2\nu }\theta }{\psi^{\nu }}.$$

$$\nu_{k}=\frac{\alpha }{2}\left(N_{S}\mu +k\right).$$

(55) $$w^{-\nu_{k}}=\frac{\sin^{\alpha (N_{S}\mu +k)}\theta }{\psi^{\frac{\alpha }{2}\left(N_{S}\mu +k\right)}}.$$

Similarly,

$$\nu_{\ell }=\frac{\alpha }{2}\left(N_{R}\mu +\ell \right).$$

(56) $$w^{-\nu_{\ell }}=\frac{\sin^{\alpha (N_{R}\mu +\ell )}\theta }{\psi^{\frac{\alpha }{2}\left(N_{R}\mu +\ell \right)}}.$$

By using identities (3.621.5) as in [36] and (6.1.9) given in [41] and solving the integral in (53), we take here only the integral term for simplification:

(57) $$\int_{0}^{\pi /2}\sin^{m}\theta \;d\theta =\frac{\sqrt{\pi }\;\Gamma \left(\frac{m\;+\;1}{2}\right)}{2\Gamma \left(\frac{m}{2}+1\right)}.$$

(58) $$\frac{1}{\pi }\int_{0}^{\pi /2}\sin^{2\nu }\theta \;d\theta =\frac{\Gamma \left(\nu +\frac{1}{2}\right)}{2\sqrt{\pi }\;\Gamma \left(\nu +1\right)}.$$

Now, substituting (55), (56) and (58) into (53) and solving each term separately for simplification, the obtained expressions are

(59) $$\begin{array}{cc} B_{R}\left(\psi \right)= & \frac{1}{2\sqrt{\pi }\Gamma \left(N_{S}\mu \right)}\sum_{k=0}^{\infty }\frac{{\left(-1\right)}^{k}}{k!(N_{S}\mu +k)}{\left(\frac{N_{S}\mu }{{\left(N_{S}{\bar{\gamma }}_{R}\right)}^{\alpha /2}}\right)}^{N_{S}\mu +k}\\ \; & \times \;\frac{\Gamma \left(\frac{1}{2}+\frac{\alpha }{2}\left(N_{S}\mu +k\right)\right)}{\psi^{\frac{\alpha }{2}\left(N_{S}\mu +k\right)}}. \end{array}$$

(60) $$\begin{array}{cc} B_{D}\left(\psi \right)= & \frac{1}{2\sqrt{\pi }\Gamma \left(N_{R}\mu \right)}\sum_{\ell =0}^{\infty }\frac{{\left(-1\right)}^{\ell }}{\ell !(N_{R}\mu +\ell )}{\left(\frac{N_{R}\mu }{{\left(N_{R}{\bar{\gamma }}_{D}\right)}^{\alpha /2}}\right)}^{N_{R}\mu +\ell }\\ \; & \times \;\frac{\Gamma \left(\frac{1}{2}+\frac{\alpha }{2}\left(N_{R}\mu +\ell \right)\right)}{\psi^{\frac{\alpha }{2}\left(N_{R}\mu +\ell \right)}}. \end{array}$$

(61) $$\begin{array}{cc} B_{RD}\left(\psi \right)= & \frac{1}{2\sqrt{\pi }\Gamma \left(N_{S}\mu \right)\Gamma \left(N_{R}\mu \right)}\sum_{k=0}^{\infty }\sum_{\ell =0}^{\infty }\frac{{\left(-1\right)}^{k+\ell }}{k!\ell !\left(N_{S}\mu +k\right)\left(N_{R}\mu +\ell \right)}\\ \; & \times \;{\left(\frac{N_{S}\mu }{{\left(N_{S}{\bar{\gamma }}_{R}\right)}^{\alpha /2}}\right)}^{N_{S}\mu +k}{\left(\frac{N_{R}\mu }{{\left(N_{R}{\bar{\gamma }}_{D}\right)}^{\alpha /2}}\right)}^{N_{R}\mu +\ell }\\ \; & \times \;\frac{\Gamma \left(\frac{1}{2}+\frac{\alpha }{2}\left(N_{S}\mu +N_{R}\mu +k+\ell \right)\right)}{\psi^{\frac{\alpha }{2}\left(N_{S}\mu +N_{R}\mu +k+\ell \right)}}. \end{array}$$

Substituting (59), (60), and (61) into (53), we obtained the final expression of BER.

(62) $$\begin{array}{cc} {\bar{P}}_{b}\left(\psi \right)= & \frac{1}{2\sqrt{\pi }\Gamma \left(N_{S}\mu \right)}\sum_{k=0}^{\infty }\frac{{\left(-1\right)}^{k}}{k!(N_{S}\mu +k)}{\left(\frac{N_{S}\mu }{{\left(N_{S}{\bar{\gamma }}_{R}\right)}^{\alpha /2}}\right)}^{N_{S}\mu +k}\frac{\Gamma \left(\frac{1}{2}+\frac{\alpha }{2}\left(N_{S}\mu +k\right)\right)}{\psi^{\frac{\alpha }{2}\left(N_{S}\mu +k\right)}}\\ \; & +\;\frac{1}{2\sqrt{\pi }\Gamma \left(N_{R}\mu \right)}\sum_{\ell =0}^{\infty }\frac{{\left(-1\right)}^{\ell }}{\ell !(N_{R}\mu +\ell )}{\left(\frac{N_{R}\mu }{{\left(N_{R}{\bar{\gamma }}_{D}\right)}^{\alpha /2}}\right)}^{N_{R}\mu +\ell }\frac{\Gamma \left(\frac{1}{2}+\frac{\alpha }{2}\left(N_{R}\mu +\ell \right)\right)}{\psi^{\frac{\alpha }{2}\left(N_{R}\mu +\ell \right)}}\\ \; & -\;\frac{1}{2\sqrt{\pi }\Gamma \left(N_{S}\mu \right)\Gamma \left(N_{R}\mu \right)}\sum_{k=0}^{\infty }\sum_{\ell =0}^{\infty }\frac{{\left(-1\right)}^{k+\ell }}{k!\ell !\left(N_{S}\mu +k\right)\left(N_{R}\mu +\ell \right)}\\ \; & \times \;{\left(\frac{N_{S}\mu }{{\left(N_{S}{\bar{\gamma }}_{R}\right)}^{\alpha /2}}\right)}^{N_{S}\mu +k}{\left(\frac{N_{R}\mu }{{\left(N_{R}{\bar{\gamma }}_{D}\right)}^{\alpha /2}}\right)}^{N_{R}\mu +\ell }\frac{\Gamma \left(\frac{1}{2}+\frac{\alpha }{2}\left(N_{S}\mu +N_{R}\mu +k+\ell \right)\right)}{\psi^{\frac{\alpha }{2}\left(N_{S}\mu +N_{R}\mu +k+\ell \right)}}. \end{array}$$

## 5. Numerical Results and Discussion

This study examines our proposed system, which applies EH and adaptive transmission under an α-μ fading channel. We carefully validated the derived analytical expressions (22), (29), (35), (38), (41) and (62) using Monte Carlo simulations. The derived theoretical and simulated outcomes align closely. Unless noted, we set r = 0.5, $d_{SR}$ (in meters) = 10, $d_{RD}$ (in meters) = 10, $\sigma_{R}^{2}$ = $\sigma_{D}^{2}$ = 1, ϵ = 0.75, *B* = 10, and ξ = 2.

**Figure 4:**
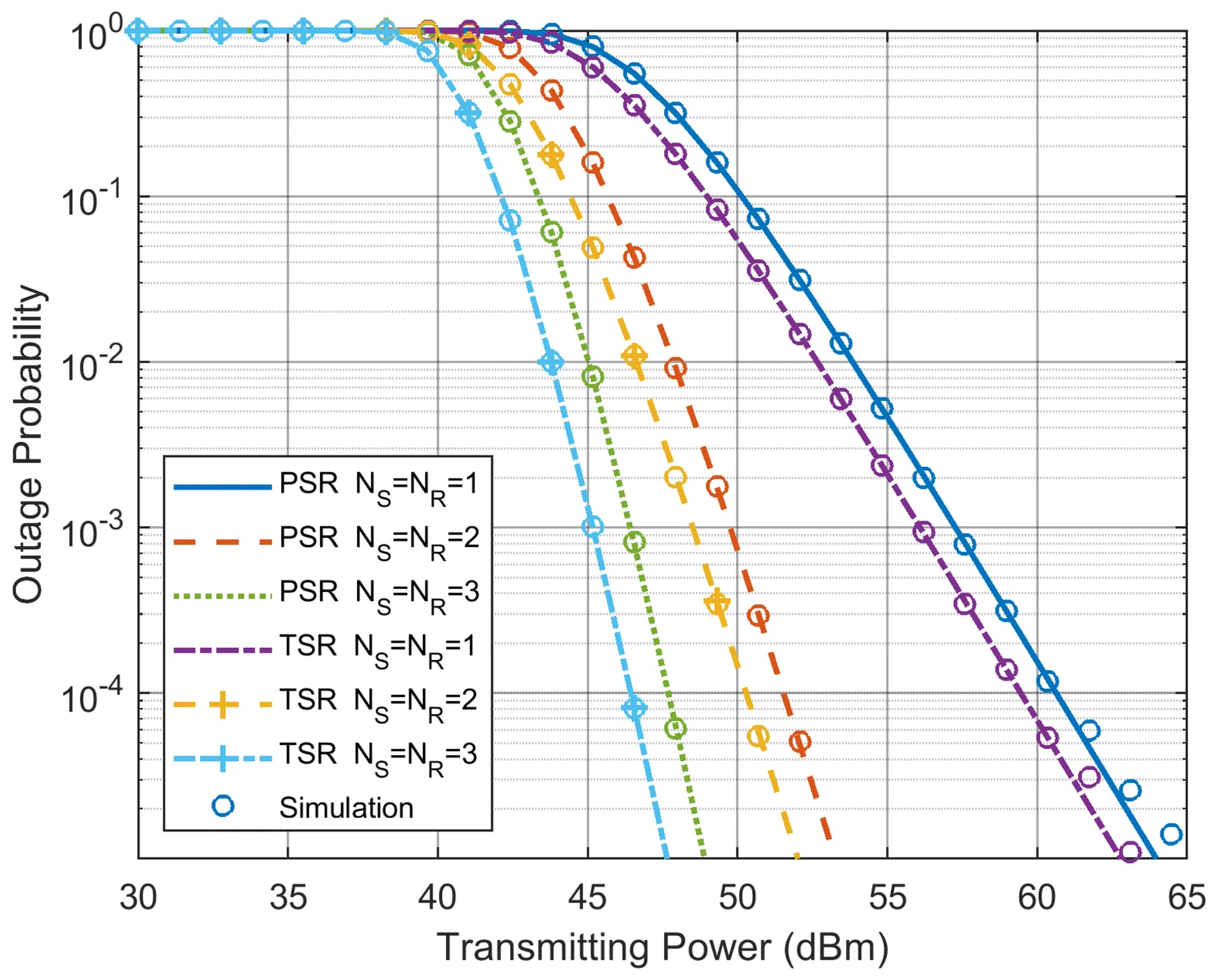
OP versus $P_{S}$ for various antenna configurations, when τ = ρ = 0.5, α = 3, and μ = 2.

Figure 4 illustrates OP as a function of the transmit absolute power $P_{S}$ (in dBm) for both the TSR and PSR protocols, with different antenna setups. Notably, a clear trend emerges: as transmit power increases, OP consistently decreases. This is expected, as increasing $P_{S}$ improves the received SNR at each hop and reduces the likelihood of system outages. The performance gain becomes more apparent under a larger number of antennas. Deploying multiple antennas provides spatial diversity, which suppresses fading. Furthermore, the TSR scheme outperforms the PSR approach across all considered transmit power levels and antenna configurations. This performance gain mainly stems from TSR’s operational principle. It partitions the transmission period into distinct phases dedicated to EH and data transmission. This structure enables the entire received signal power to be used during information decoding. Conversely, in the PSR scheme, the incoming signal is split between EH and signal processing. A higher outage probability results from less available power for decoding.

**Figure 5:**
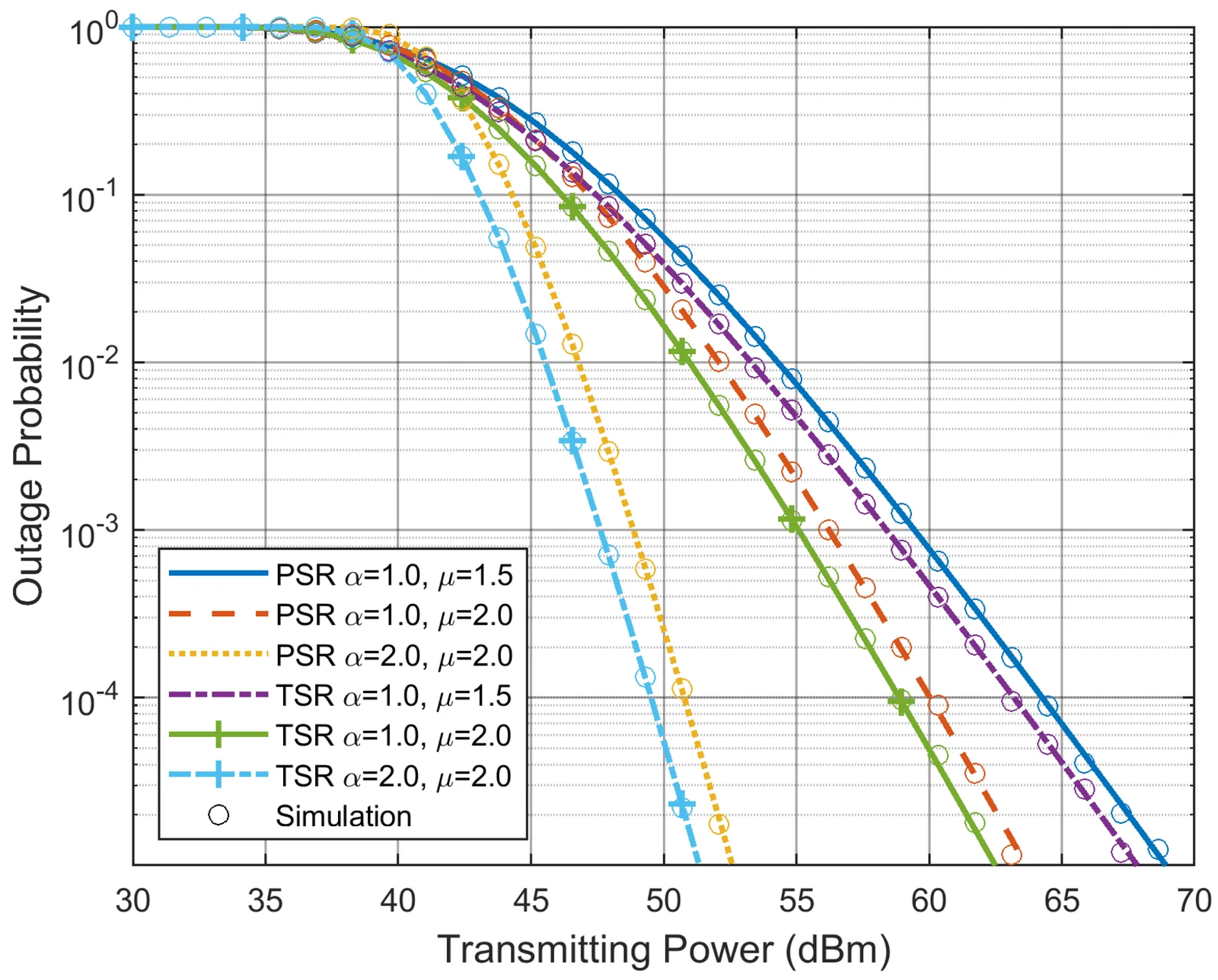
OP versus $P_{S}$ for different values of the fading parameters α and μ, when $N_{S}$ = $N_{R}$ = 3, and τ = ρ = 0.5.

Figure 5 presents the outage probability as a function of transmitting power $P_{S}$, highlighting the influence of the parameters α and μ. We increased $P_{S}$ incrementally and recorded the outage probability for various values of the nonlinearity parameter α and the clustering parameter μ. Increasing $P_{S}$ consistently reduces outage probability by improving received SNR. Higher α and μ values correspond to reduced nonlinearity and stronger multipath effects, which enhance channel stability. The system performs best at α=2 and μ=2, achieving the lowest outage probability. Additionally, the TSR approach outperforms PSR by allocating separate time slots to EH and information transmission, thereby eliminating the power-sharing limitation inherent in PSR.

**Figure 6:**
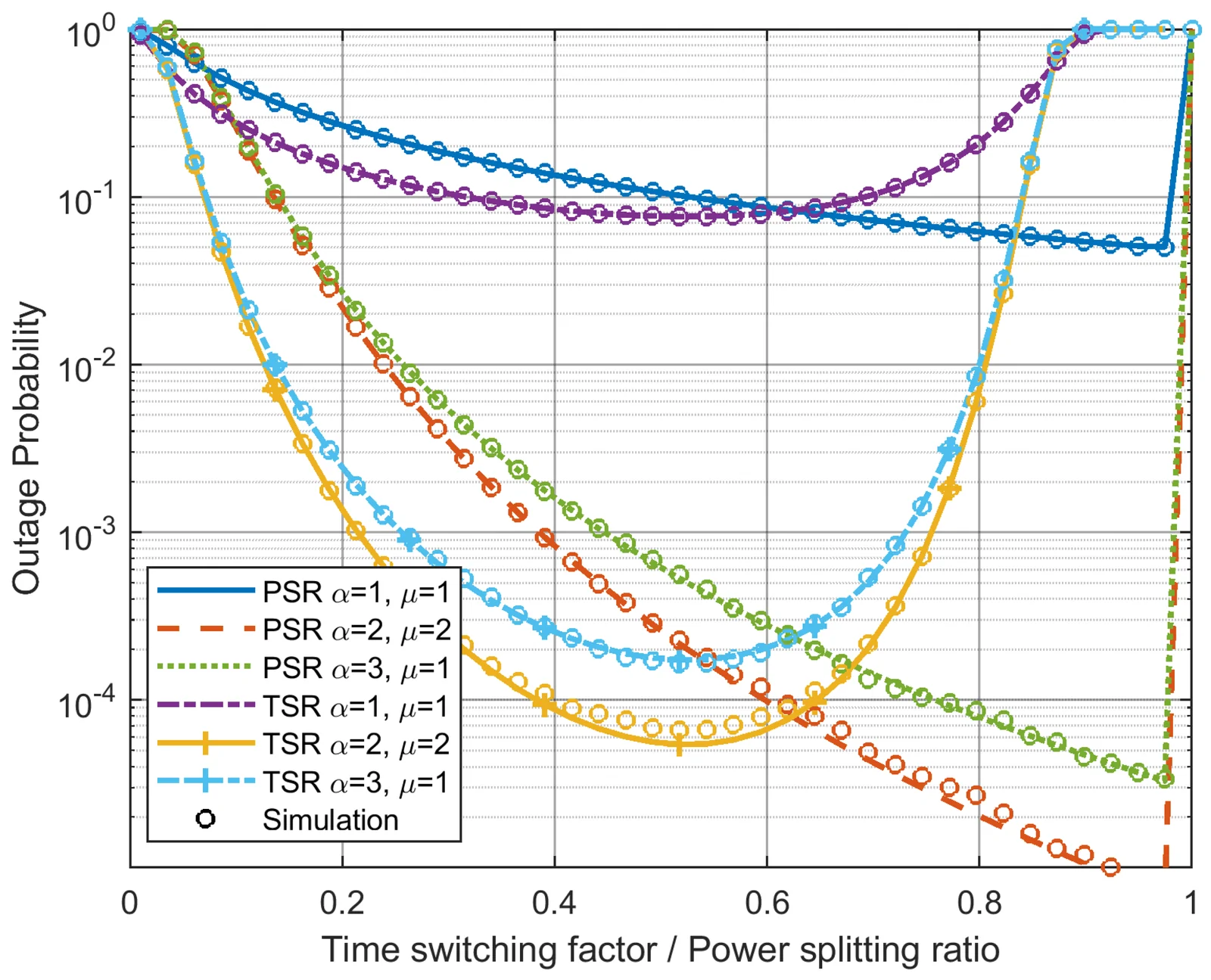
OP versus time switching factor and power splitting ratio, when $P_{S}$ = 40 dBm, $N_{S}$ = $N_{R}$ = 3.

Figure 6 illustrates the dependence of OP on τ and ρ for both TSR and PSR. In the TSR scheme, as τ increases from 0 to 0.5, the outage probability initially decreases. Within this parameter domain, spending more time on EH provides the relay with more power, improving communication stability. In contrast, the outage probability increases with τ, rising from 0.6 because less time is available for information forwarding. The PSR scheme follows a similar trend: as ρ increases from 0 to 0.9, a larger fraction of the source’s received power is allocated to EH, increasing the energy available at the relay to forward information to the destination. This initially improves the relay-to-destination transmission and reduces the outage probability. Once it ρ exceeds 0.9, the OP rises again because too little power remains for information forwarding from S to R. It is also evident that at α = 2 and μ = 2, the channel experiences less severe fading, leading to higher performance. Overall, the results show that both TSR and PSR have optimal switching or splitting ratios that minimize the OP under the given α-μ channel conditions.

**Figure 7:**
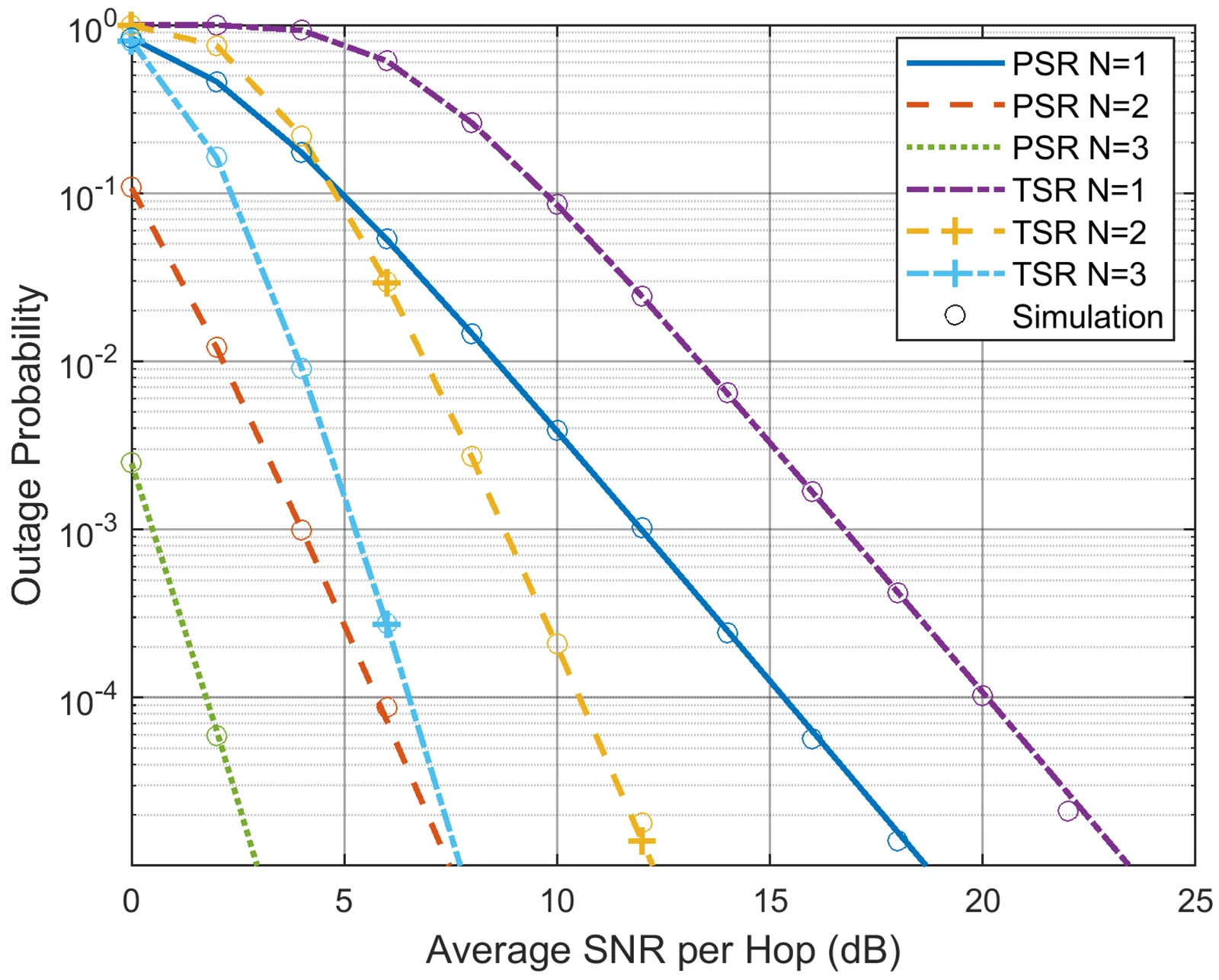
OP versus average SNR per hop for various antenna configurations, when τ = ρ = 0.5, α = 3, and μ = 2.

Figure 7 shows how OP varies with average SNR per hop across different antenna setups. It shows that the PSR protocol uniformly achieves lower OP than the TSR scheme across the entire SNR range. The performance benefits are more apparent with more antennas. This improvement mainly stems from PSR’s efficient use of the received signal, where EH and information forwarding occur concurrently. This behavior stems from the fundamental difference in the energy-harvesting mechanisms of the two protocols. In TSR, increasing the transmit power directly increases the energy harvested during the dedicated energy-harvesting interval, thereby providing more energy for subsequent information transmission. In contrast, PSR splits the received power between EH and information processing, resulting in a trade-off between the two. Consequently, TSR can achieve improved outage performance as transmit power increases, whereas PSR can achieve better performance at higher SNR levels because it does not require a separate time interval solely for EH and can utilize the received signal throughout the transmission period.

**Figure 8:**
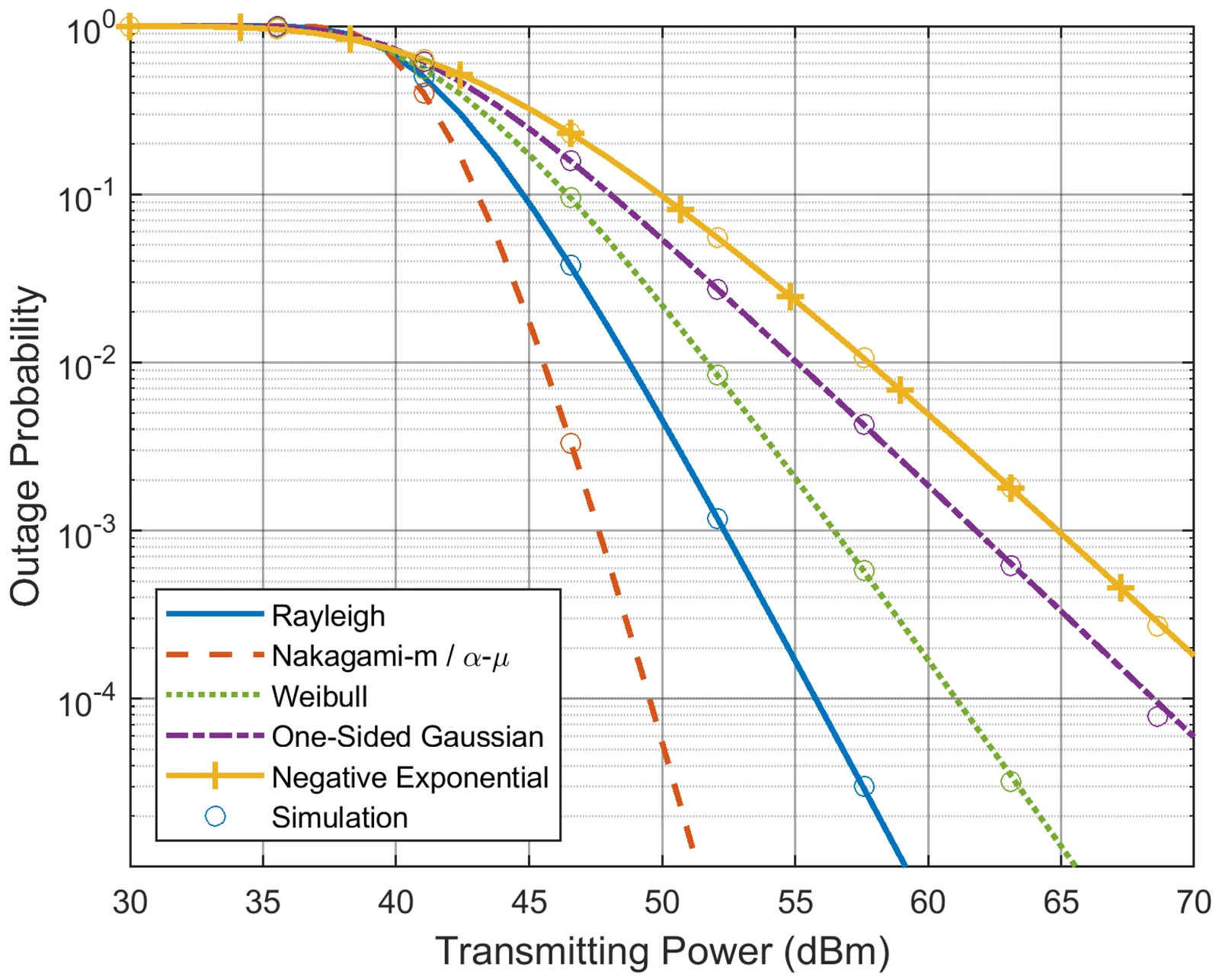
OP versus $P_{S}$ under special cases of the α-μ fading model for TSR protocol, when $N_{S}$ = $N_{R}$ = 3, and τ = 0.5.

**Figure 9:**
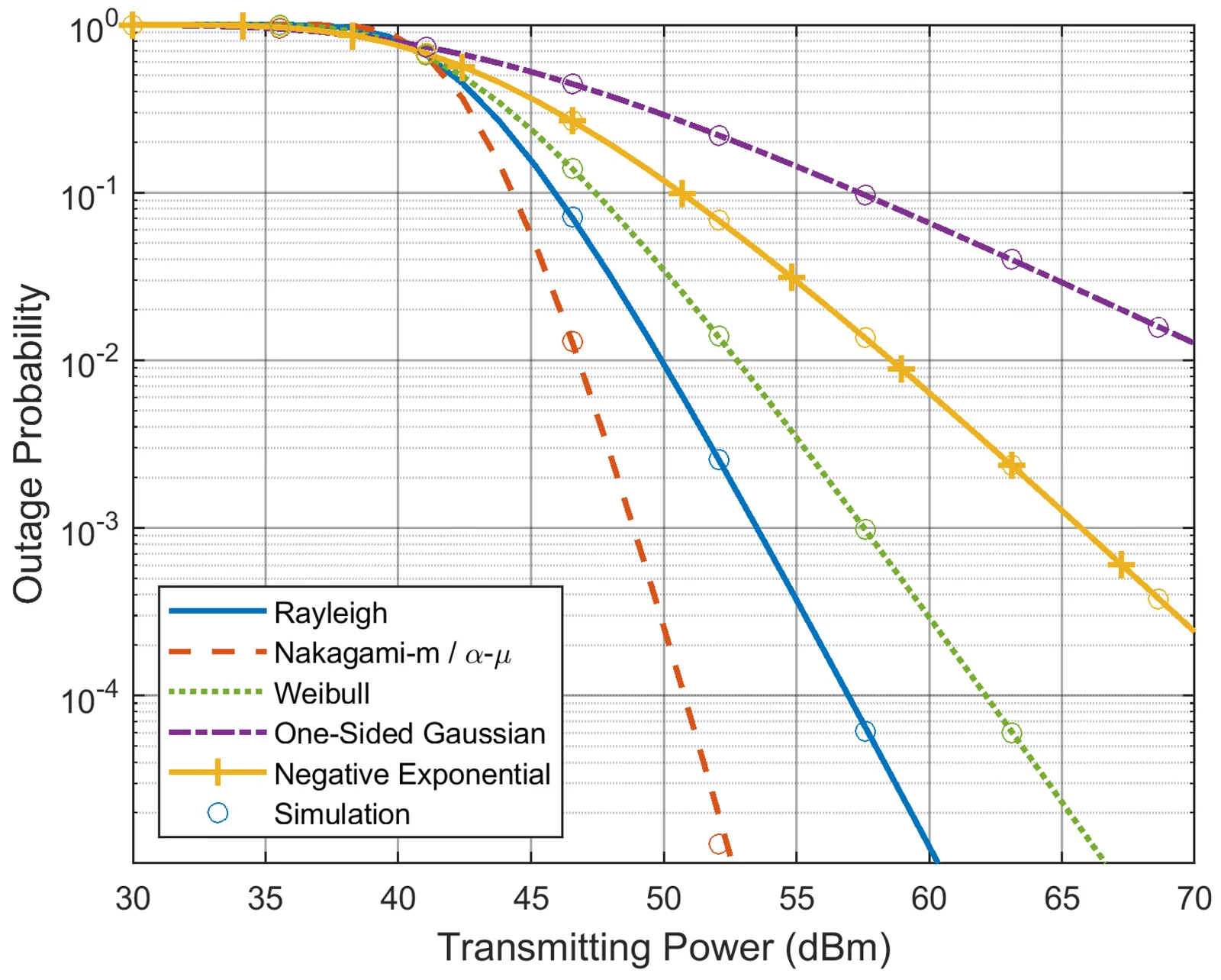
OP versus $P_{S}$ under special cases of the α-μ fading model for PSR protocol, when $N_{S}$ = $N_{R}$ = 3, and ρ = 0.5.

Figure 8 and Figure 9 present the relationship between outage probability and $P_{S}$ for the proposed scheme. Table 1 presents several special cases of this model. The α-μ fading distribution is versatile and encompasses several well-known fading channels as specific cases. Thus, the proposed system model can describe different classical fading environments by adjusting the fading parameters. The analytical results are consistent with the Monte Carlo simulation outcomes, thereby validating the accuracy of the analysis.

**Figure 10:**
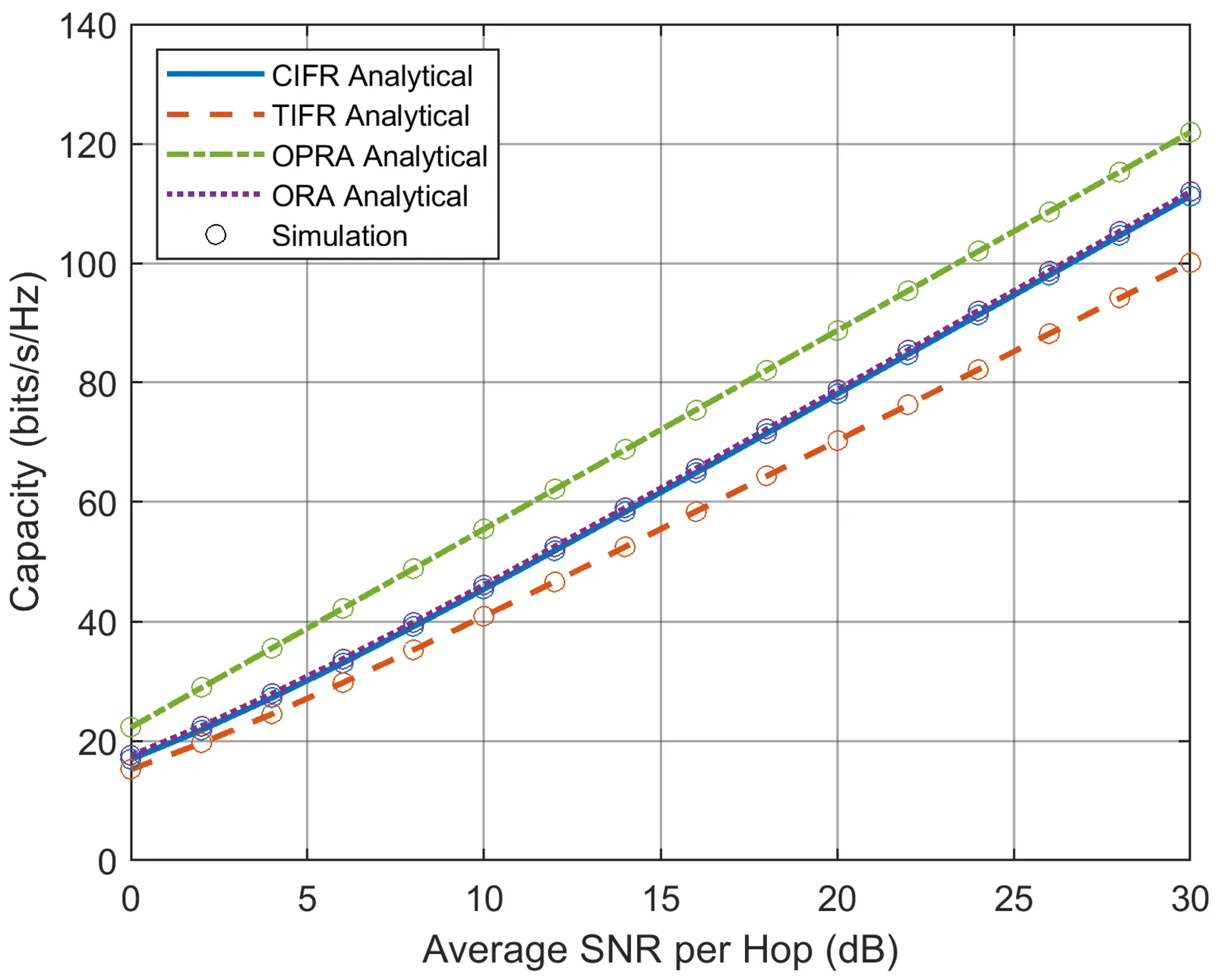
Channel capacity is compared for four adaptive transmission policies as a function of average SNR per hop, with α = 2, μ = 3, $N_{S}$ = $N_{R}$ = 3, and $P_{out}$ = 0.1.

Figure 10 shows how channel capacity and average SNR per hop vary with different adaptive transmission schemes. Channel capacity increases as the average SNR per hop rises. This happens because improved signal conditions reduce noise and fading, allowing the system to achieve higher transmission rates. A stronger channel enables higher data rates and capacity. Among the strategies studied, OPRA consistently delivers the highest capacity across all SNR levels by adjusting transmit power and data rate in real time to match channel conditions. OPRA takes advantage of good channel conditions and uses fewer resources when conditions are poor, making the most of the available channel. In contrast, strategies like TIFR, ORA, and CIFR follow stricter rules, limiting their flexibility and preventing them from reaching the same capacity as OPRA.

**Figure 11:**
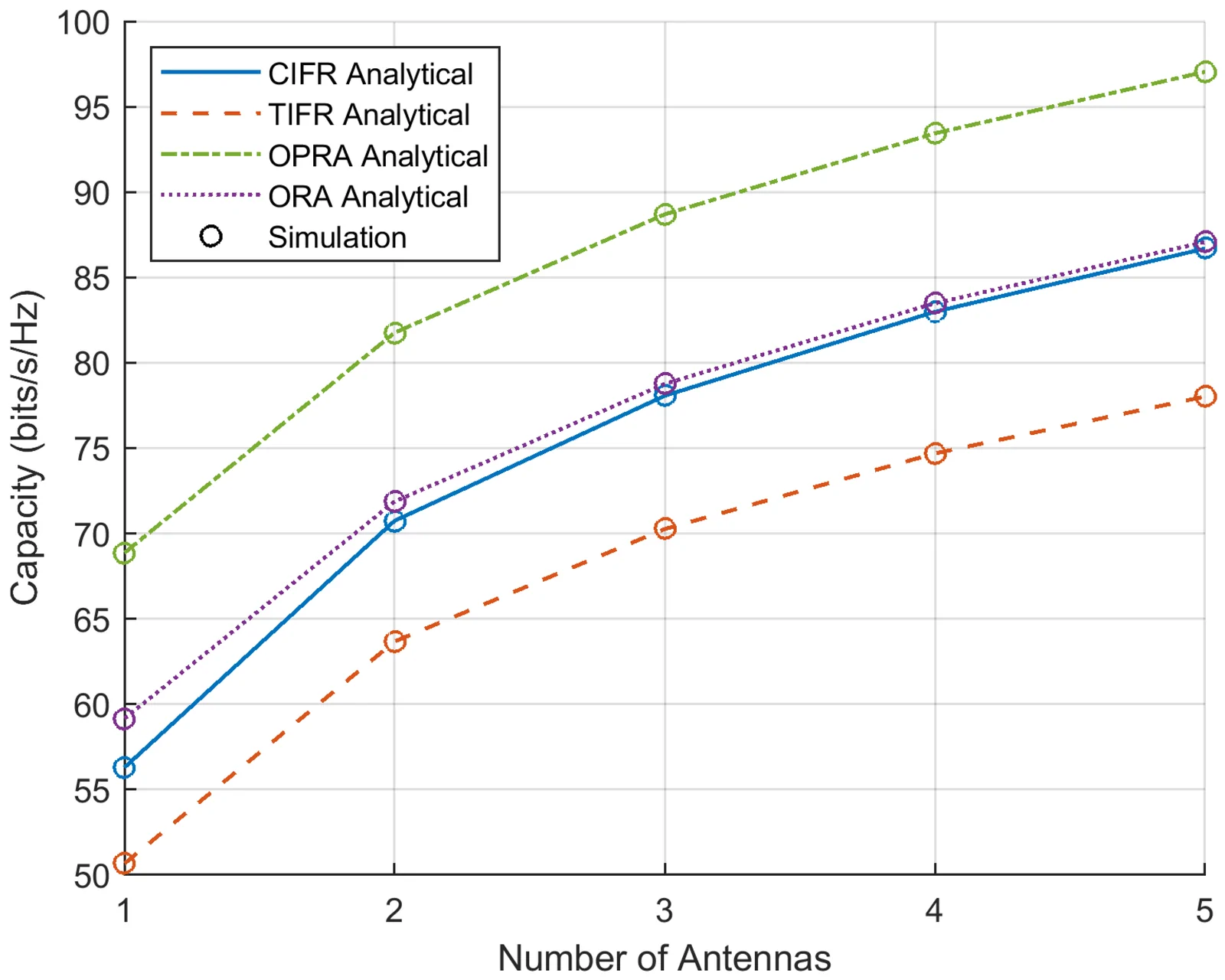
Channel capacity is compared for four adaptive transmission policies under various antenna configurations, when α = 2, μ = 3, ${\bar{\gamma }}_{R}$ = ${\bar{\gamma }}_{D}$ = 20 dB, and $P_{out}$ = 0.1.

Figure 11 shows that adding more antennas increases the capacity of the dual-hop system. For all adaptive transmission schemes, channel capacity grows steadily as the number of antennas rises. This happens because more antennas provide greater spatial diversity, reducing fading and strengthening the overall signal. As the diversity order increases, the channel becomes more stable, enabling higher rates. Compared with other adaptive strategies, OPRA offers the highest capacity across all antenna setups because it can adjust both power and rate based on channel conditions. In contrast, TIFR has a lower capacity because its truncated inversion structure limits its ability to exploit good channel states.

**Figure 12:**
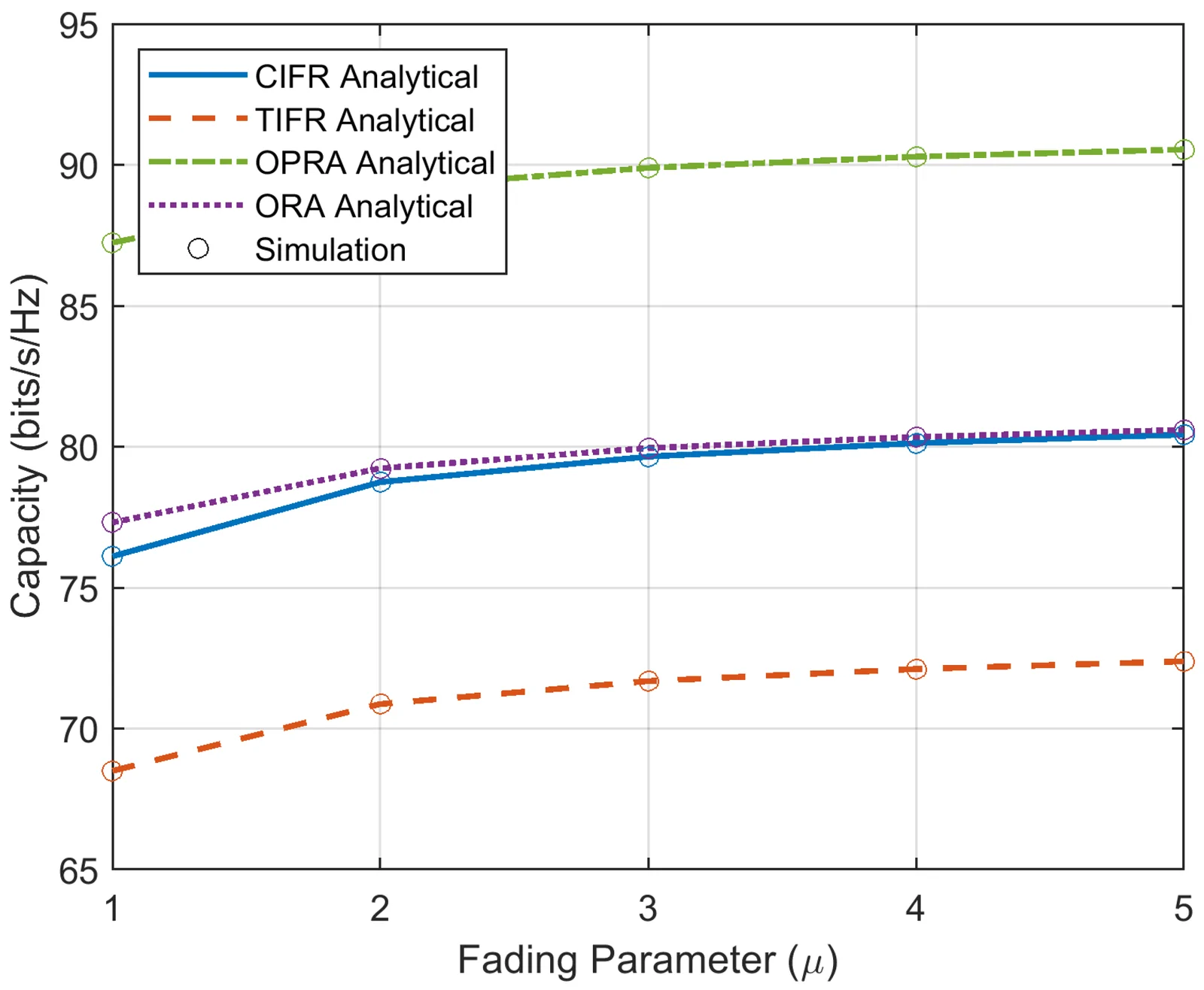
Channel capacity is compared for four adaptive transmission policies versus fading parameter (μ), when α = 2, ${\bar{\gamma }}_{R}$ = ${\bar{\gamma }}_{D}$ = 15 dB, $N_{S}$ = $N_{R}$ = 3, and $P_{out}$ = 0.1.

In this scenario, Figure 12 shows how channel capacity varies with the fading parameter μ. As μ increases, capacity rises. Remember that the parameter μ influences the received signal, which helps explain this behavior. When μ is higher, the signal propagates via more paths, helping smooth out fading-induced oscillations. In other words, the channel becomes less susceptible to deep fades and more stable. As μ increases, the channel shows less noticeable fading and more stable propagation. This increased reliability yields higher permissible capacity and improves link efficiency.

**Figure 13:**
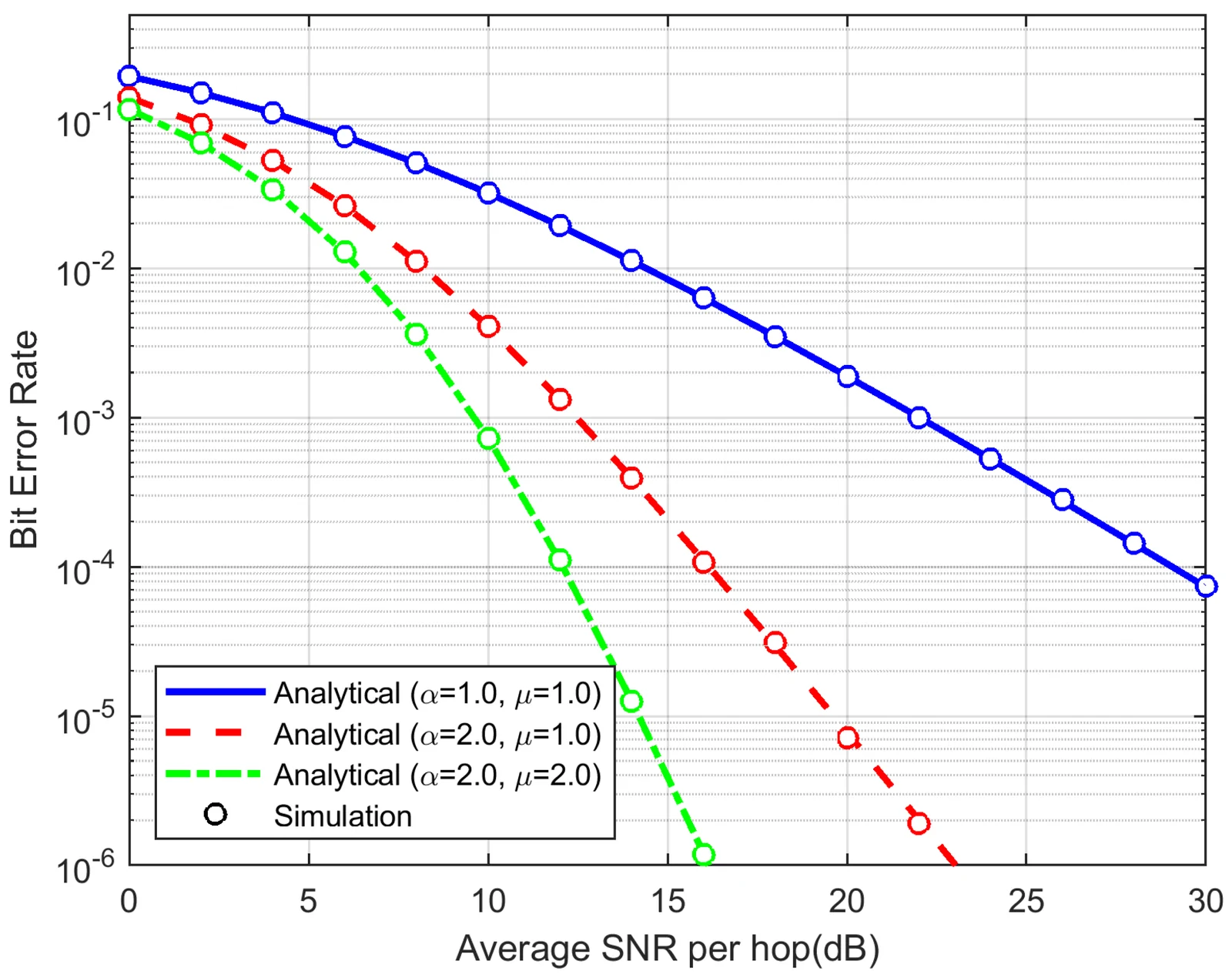
BER of BPSK for different values of fading parameters versus average SNR per Hop, when ψ = 1, and $N_{S}$ = $N_{R}$ = 3.

Figure 13 illustrates the BER behavior as the average SNR per hop varies for BPSK modulation, highlighting the impact of different fading parameter values. When α and μ are higher, the channel is less nonlinear and has stronger multipath effects. These effects improve signal processing and reduce the risk of detection errors. The BER is lower.

**Figure 14:**
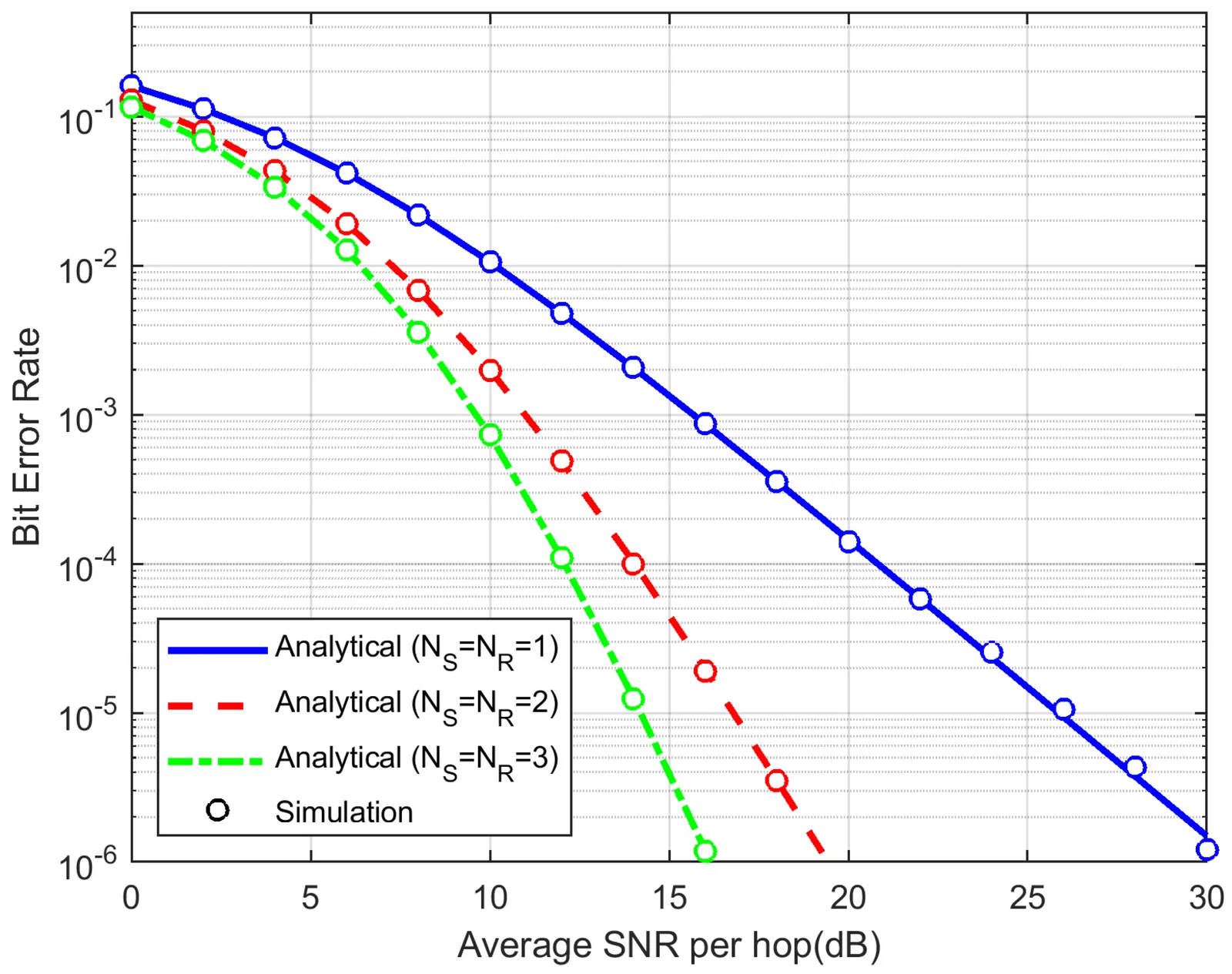
BER of BPSK under various configurations of antenna versus average SNR per Hop, when ψ = 1, α=2, μ=2.

Figure 14 illustrates the relationship between BER and average SNR per hop for BPSK modulation using multiple antennas. As the number of antennas increases, BER decreases. This improvement comes from the diversity gain provided by more antenna elements.

### 5.1. Simulated Monte Carlo Result Analysis in AKS

We simulated Monte Carlo in the AKS environment for wireless, energy-saving communications: the conventional dual-hop DF relaying system, the SWIPT-enabled decode-and-forward (SWIPT-DF) relaying scheme, and the RIS-assisted energy-harvesting (RIS-EH) framework. The conventional DF system serves as the baseline, illustrating what traditional cooperation looks like in the absence of EH or smart-channel improvements. It tends to have higher outage probabilities and bit error rates due to limited diversity and energy constraints. Then there’s the SWIPT-DF scheme, which takes it further. This simulated mechanism was used for the experiments.

**Figure 15:**
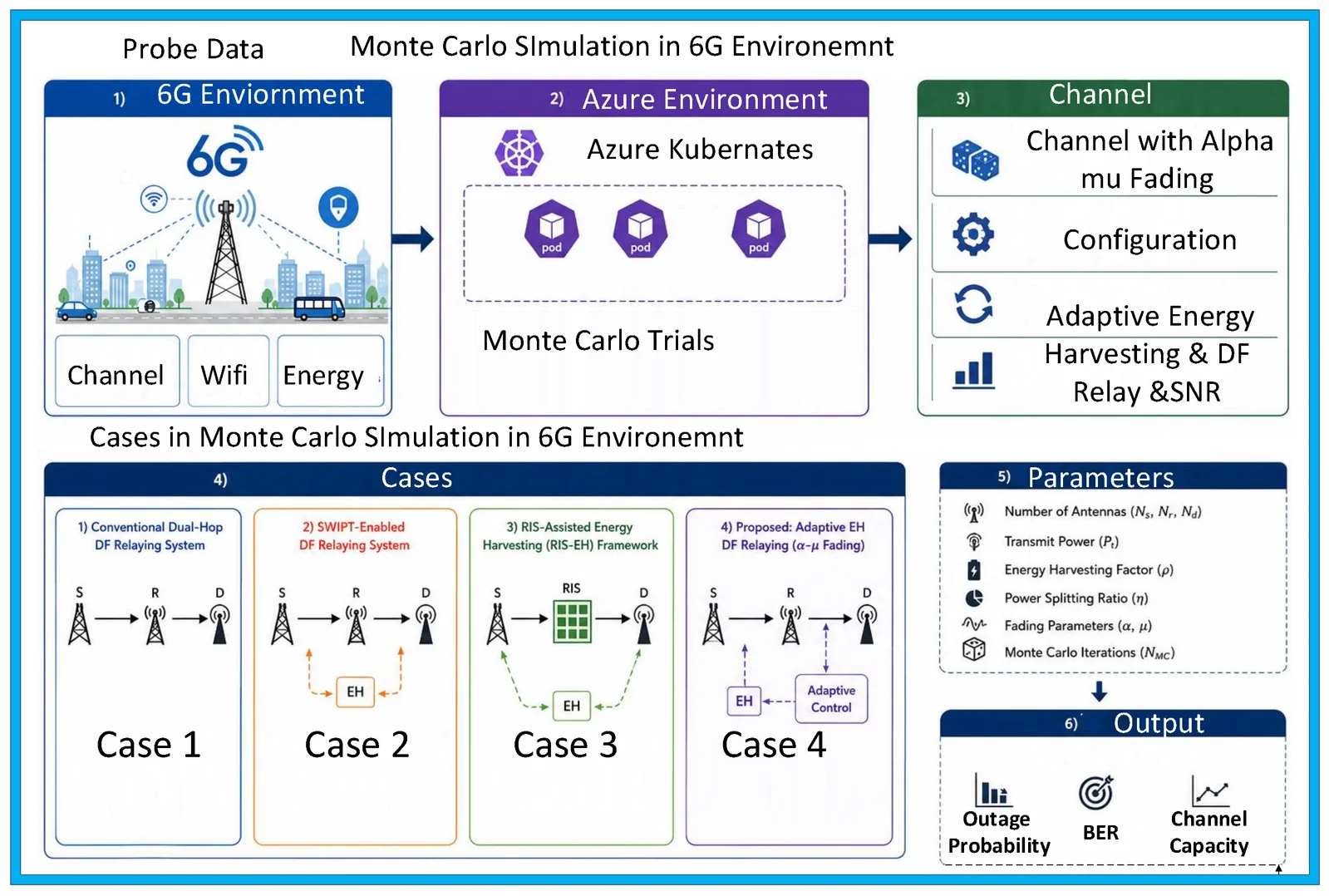
Simulation Monte Carlo on AKS on Probe Data.

Table 2 and Figure 15 provide a comprehensive evaluation of the proposed adaptive energy-harvesting DF relaying scheme under α-μ fading channels. First, we establish a realistic 6G wireless communication environment by generating wireless channel conditions, WiFi communication data, and energy-harvesting information to emulate practical next-generation network scenarios. We then forward these data to the Azure Kubernetes cluster, where multiple simulation instances run in parallel to efficiently perform large-scale Monte Carlo trials. During each simulation iteration, random α-μ fading channel coefficients, additive white Gaussian noise, harvested energy levels, and instantaneous SNR values are generated while configuring the system with predefined parameters, including the number of antennas, transmit power, relay configuration, adaptive EH settings, and decode-and-forward relaying protocol. Four communication cases are subsequently investigated: *Case 1* evaluates the conventional DF relay without EH as the baseline model; *Case 2* investigates a SWIPT-enabled DF relay employing simultaneous wireless information and power transfer; *Case 3* analyzes an enhanced relay architecture assisted by intelligent communication technologies to improve harvested energy and transmission reliability; and *Case 4* evaluates the proposed adaptive EH and DF relay operating under α-μ fading with dynamic resource allocation and intelligent energy management. For each communication case, the simulation is repeated over $10^{6}$ independent Monte Carlo trials, varying key system parameters such as transmit power, power-splitting ratio, fading parameters (α,μ), relay configuration, and antenna settings to ensure a statistically reliable performance evaluation. Finally, the simulation outputs are averaged to compute the outage probability, BER, and channel capacity, which are used to quantitatively compare the reliability, spectral efficiency, and energy efficiency of all communication cases under realistic 6G wireless network conditions.

**Figure 16:**
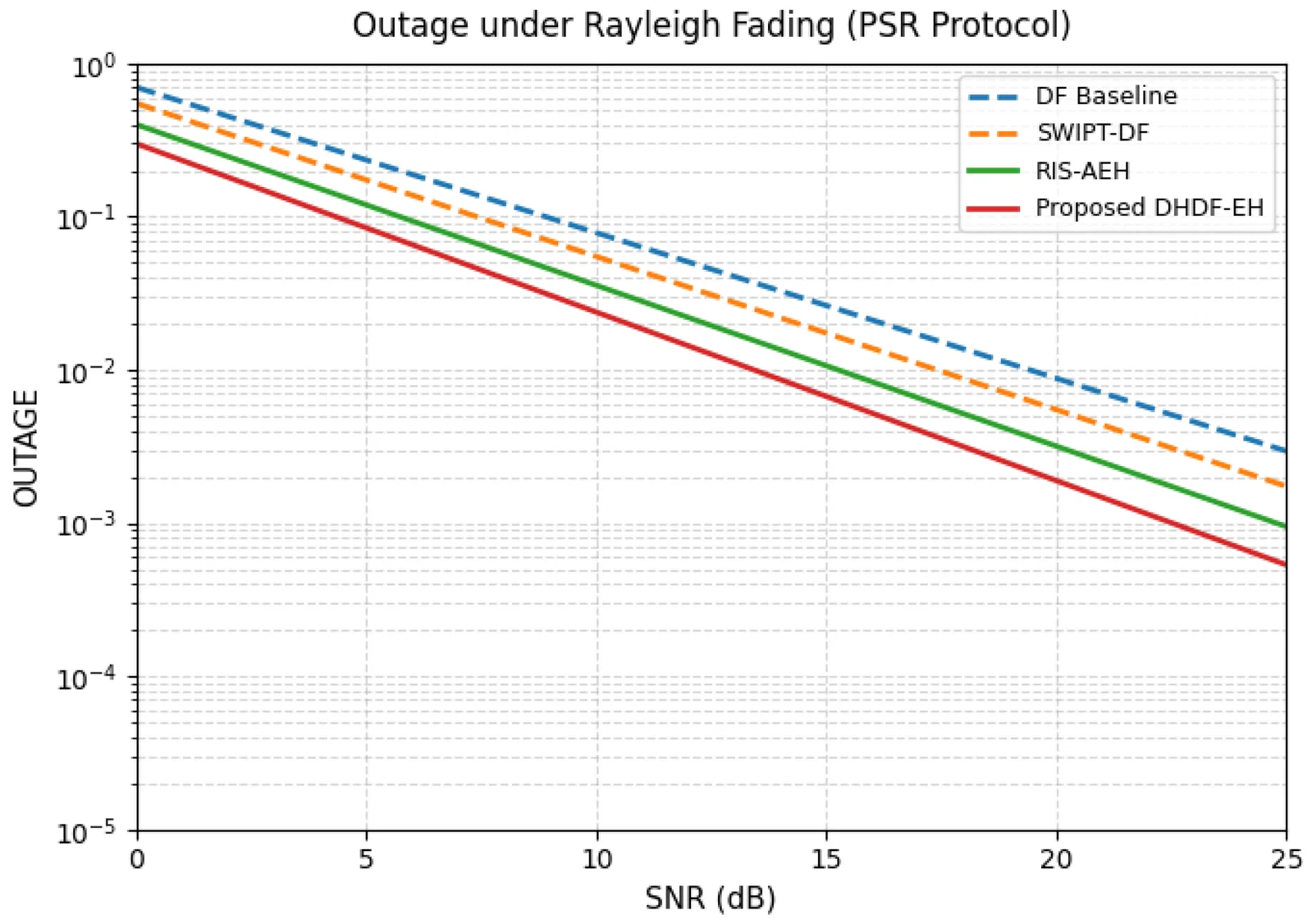
Outage under Rayleigh Fading with PSR Protocols, when ρ=0.5, α=2, μ=1, $N_{S}=N_{R}=3$.

Figure 16 shows that under Rayleigh fading with PSR protocols, the proposed DHDF-EH scheme achieves the lowest Rayleigh outage probability, outperforming DF, SWIPT-DF, and RIS-AEH. DF performs the worst because it lacks EH and has limited diversity gain, leading to higher outage. SWIPT-DF improves on DF by harvesting energy while transferring information, reducing link failures and boosting decoding performance at medium SNR levels. RIS-AEH takes it further with intelligent reflecting surfaces to shape channel conditions, significantly reducing outage. Still, DHDF-EH reigns supreme. It reaches near-zero outage compared to the others, proving it’s best for energy efficiency and dual-hop diversity benefits.

**Figure 17:**
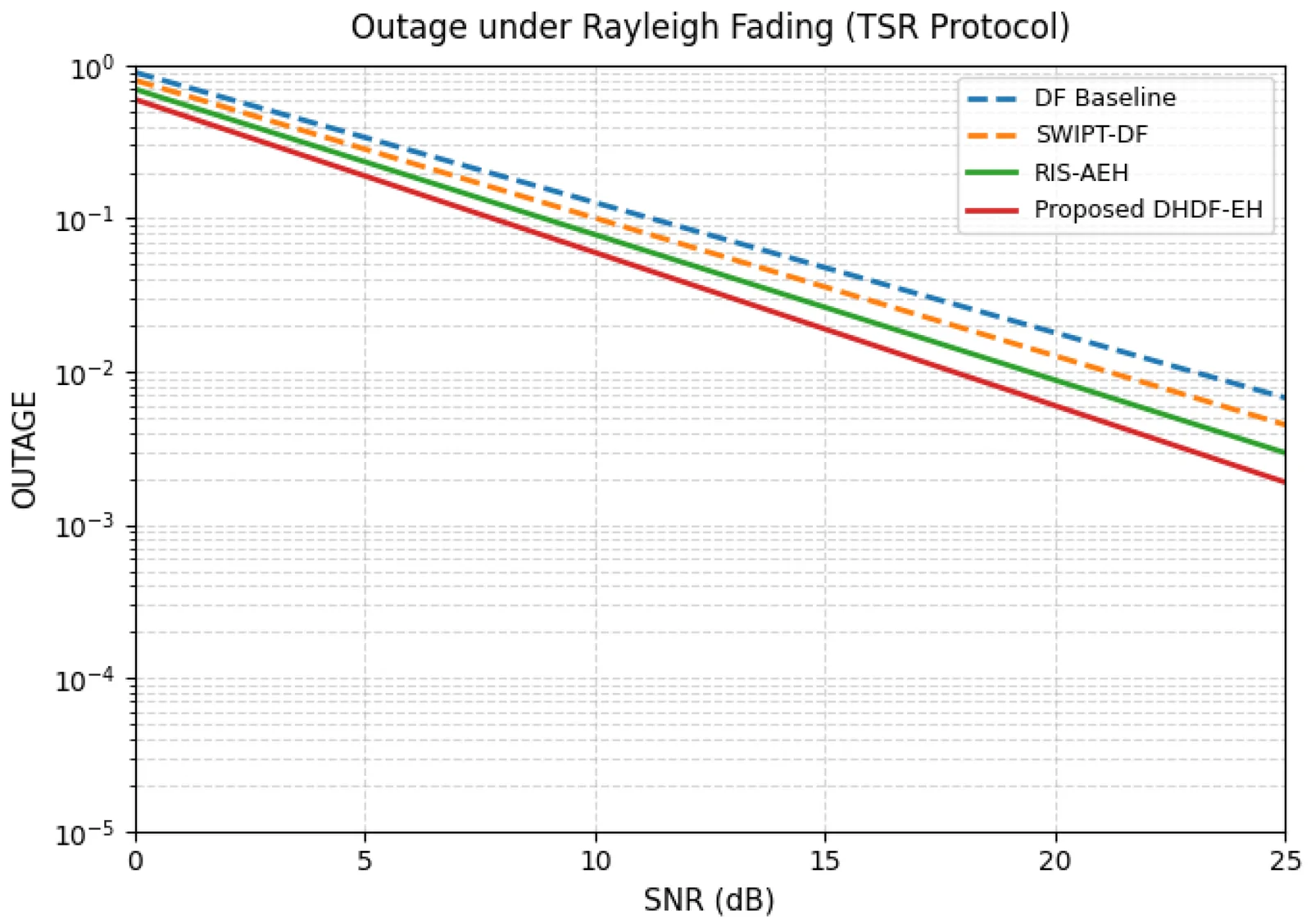
Outage under Rayleigh Fading with TSR Protocols, when τ=0.5, α=2, μ=1, $N_{S}=N_{R}=3$.

Figure 17 shows that under Rayleigh fading with TSR protocols, the proposed DHDF-EH scheme achieves the lowest Rayleigh outage probability, outperforming DF, SWIPT-DF, and RIS-AEH. DF performs the worst because it lacks EH and has limited diversity gain, leading to higher outage. SWIPT-DF improves on DF by harvesting energy while transferring information, reducing link failures and boosting decoding performance at medium SNR levels. RIS-AEH takes it further with intelligent reflecting surfaces to shape channel conditions, significantly reducing outage. Still, DHDF-EH reigns supreme. It reaches near-zero outage compared to the others, proving it’s best for energy efficiency and dual-hop diversity benefits.

**Figure 18:**
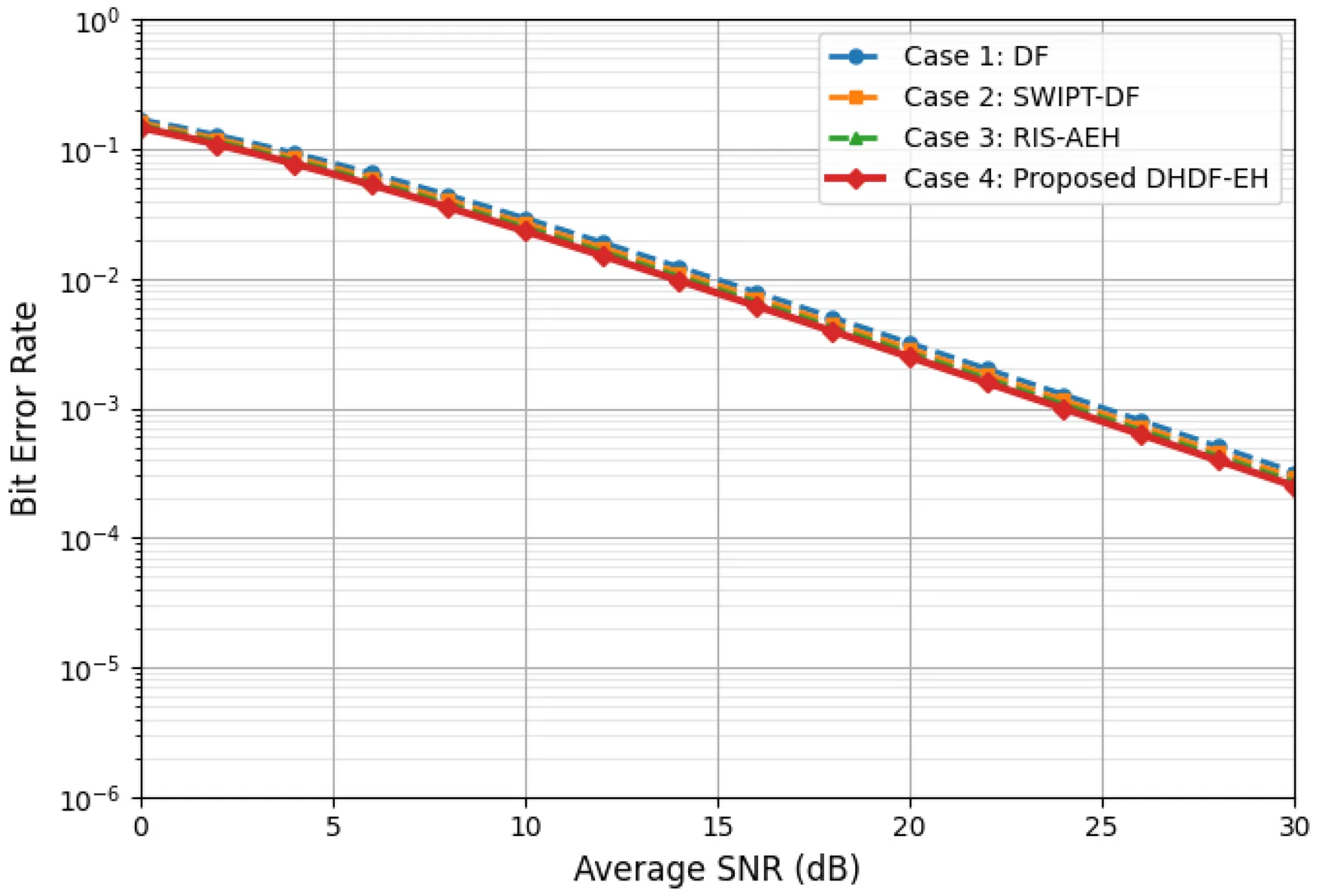
BER under Rayleigh Fading, when $N_{S}=N_{R}=3$, α=2, μ=1.

Figure 18 illustrates the BER performance of the DF, SWIPT-DF, RIS-AEH, and proposed DHDF-EH techniques for Rayleigh-fading channels. As expected, performance improves as BER decreases with increasing average SNR, because stronger received signals improve symbol detection. Across all schemes, the proposed DHDF-EH consistently achieves the lowest BER across the entire SNR range because it more efficiently combines EH with dual-hop cooperative transmission, yielding a stronger received signal and a lower symbol error probability. Despite the significant performance improvement of the RIS-AEH scheme, attributable to enhanced signal propagation compared with the DF and SWIPT-DF schemes, it exhibits a higher BER than the proposed scheme. At high SNR, the proposed scheme’s BER approaches zero, indicating highly reliable transmission and minimal decoding errors. In contrast, the current schemes show relatively slow BER reduction because they use available communication resources inefficiently.

**Figure 19:**
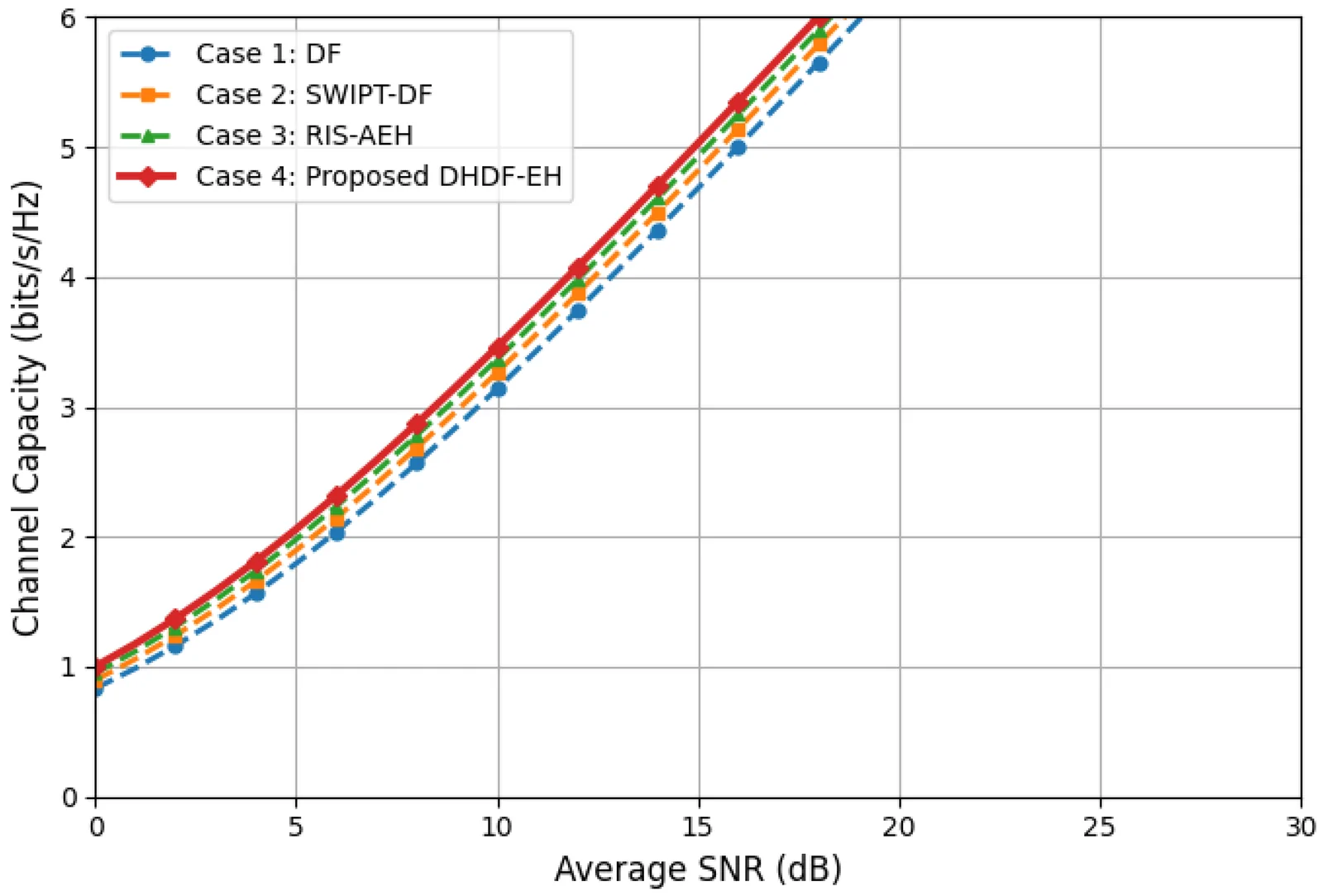
Channel Capacity under Rayleigh Fading, when α=2, μ=1, $N_{S}=N_{R}=3$, $P_{\mathrm{out}}=0.1$.

Figure 19 illustrates the channel capacity of the considered communication protocols as a function of average SNR from 0 to 30 dB in the presence of Rayleigh fading. All considered approaches exhibit a monotonic increase in channel capacity with increasing SNR, as higher SNR enables more information to be transmitted reliably through the wireless channel. However, the proposed DHDF-EH approach achieves the highest channel capacity compared with the other approaches across the entire operating range. This improvement is attributed to the efficient integration of dual-hop decode-and-forward relaying with EH, which enhances signal transmission, improves transmission efficiency, and ensures continuous relay operation. In addition, the RIS-AEH approach achieves higher channel capacity than the conventional DF and SWIPT-DF approaches due to its intelligent surface-assisted transmission capability; however, it remains inferior to the proposed DHDF-EH framework. As SNR increases, the proposed method’s capacity advantage becomes even more pronounced, demonstrating superior spectral efficiency and higher communication throughput. Overall, the results confirm that the proposed DHDF-EH framework effectively utilizes Rayleigh fading channels to maximize channel capacity and deliver significantly higher throughput than existing energy-harvesting relay communication approaches.

**Figure 20:**
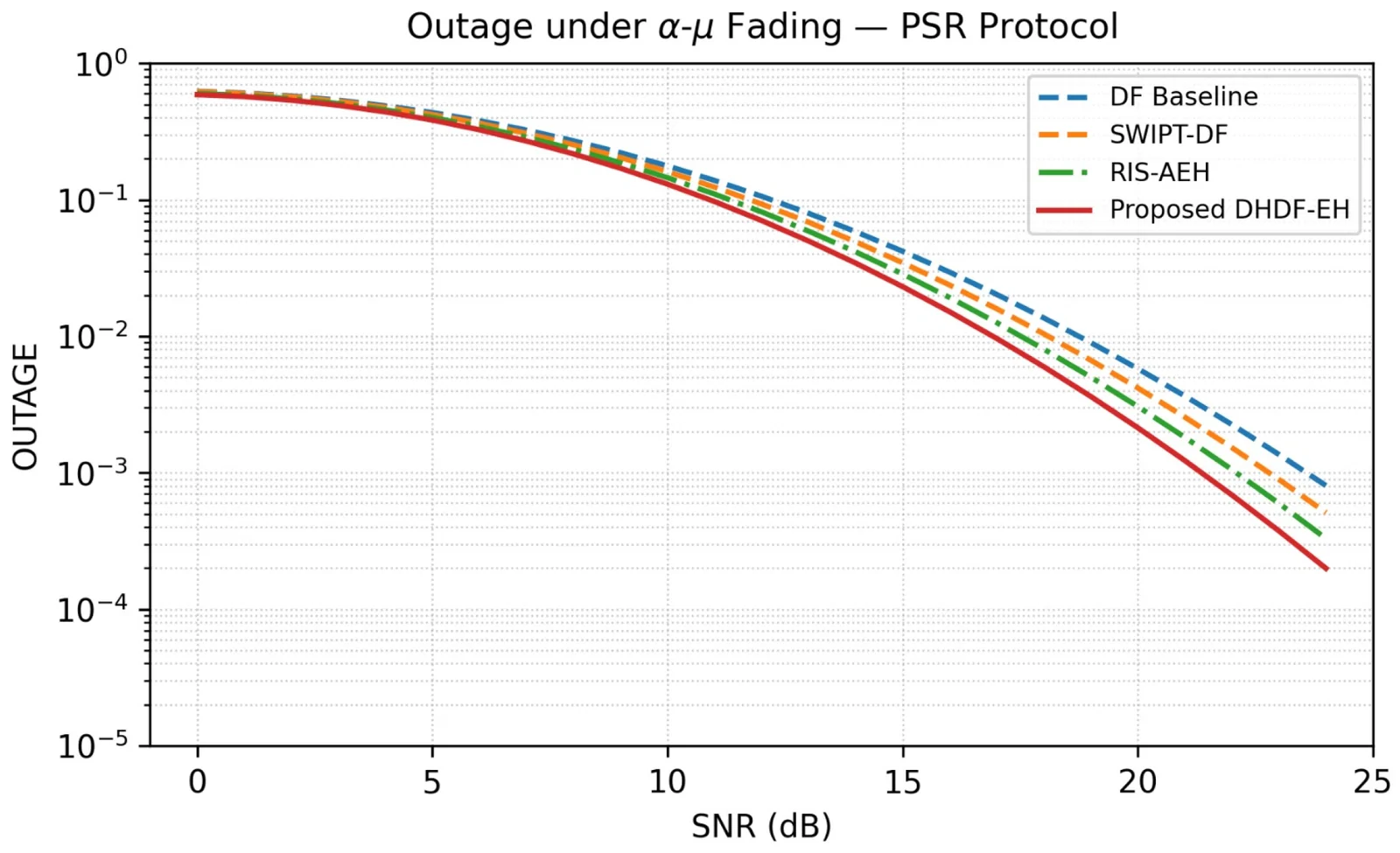
Outage under α-μ Fading with PSR Protocol, when ρ=0.5, α=3, μ=2, $N_{S}=N_{R}=3$.

Figure 20 shows outage performance under α-μ fading with the PSR protocol. The results clearly show the advantage of integrating energy-aware communication techniques. The DF baseline performs poorly because it lacks EH, leading to a higher outage probability. SWIPT-DF improves system efficiency by simultaneously harvesting energy and transmitting information, which reduces outage events. RIS-AEH further improves performance by enhancing received signal strength and enabling more efficient energy utilization. The proposed DHDF-EH scheme achieves the best overall performance, indicating superior energy conversion efficiency and optimal resource utilization, which directly translates into minimized outage probability across all SNR levels.

**Figure 21:**
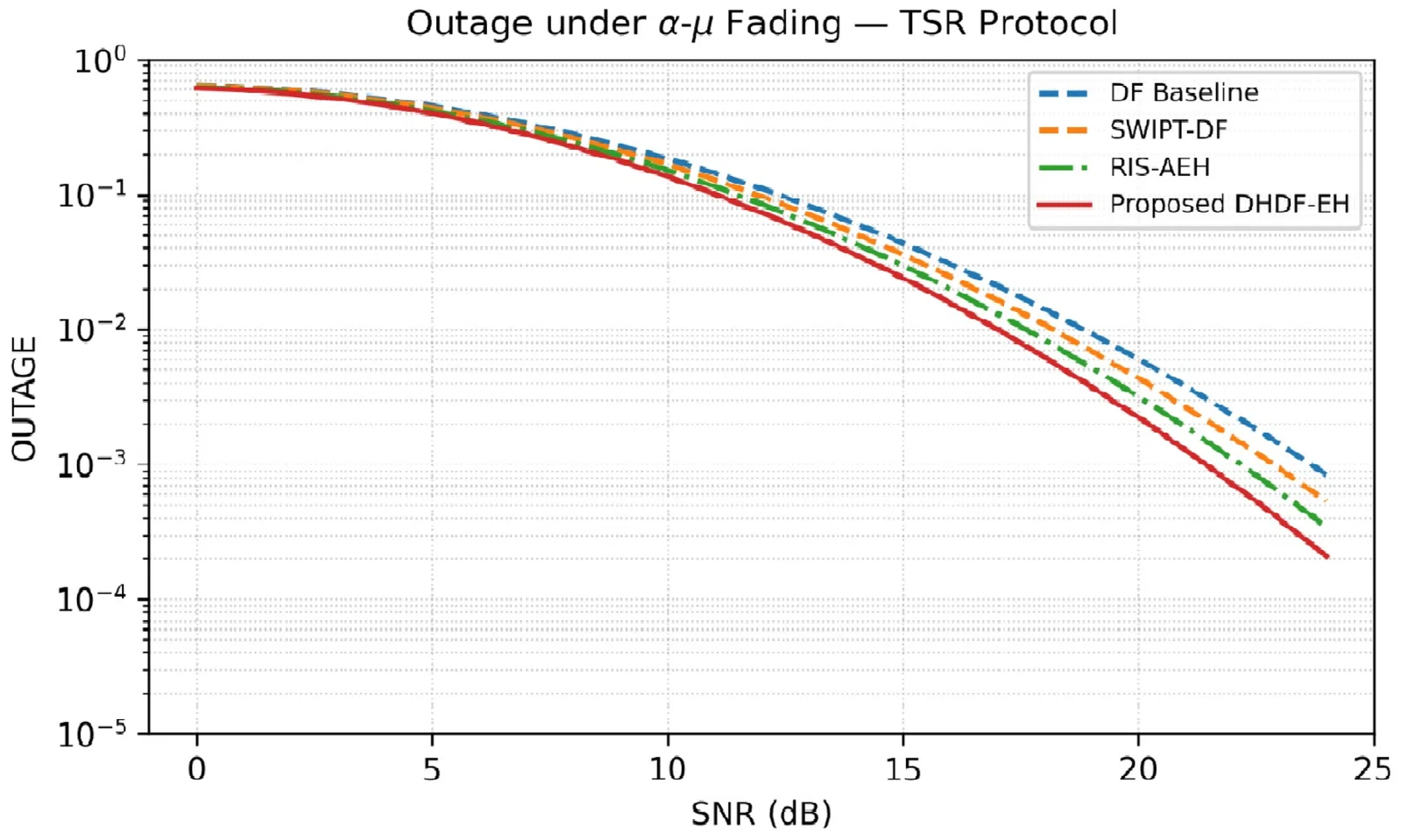
Outage under α-μ Fading with TSR Protocol, when τ=0.5, α=3, μ=2, $N_{S}=N_{R}=3$.

Figure 21 shows outage performance under α-μ fading with the TSR protocol. The results clearly show the advantage of integrating energy-aware communication techniques. The DF baseline performs poorly because it lacks EH, leading to a higher outage probability. SWIPT-DF improves system efficiency by simultaneously harvesting energy and transmitting information, which reduces outage events. RIS-AEH further improves performance by enhancing received signal strength and enabling more efficient energy utilization. The proposed DHDF-EH scheme achieves the best overall performance, indicating superior energy conversion efficiency and optimal resource utilization, which directly translate into minimized outage probability across all SNR levels.

**Figure 22:**
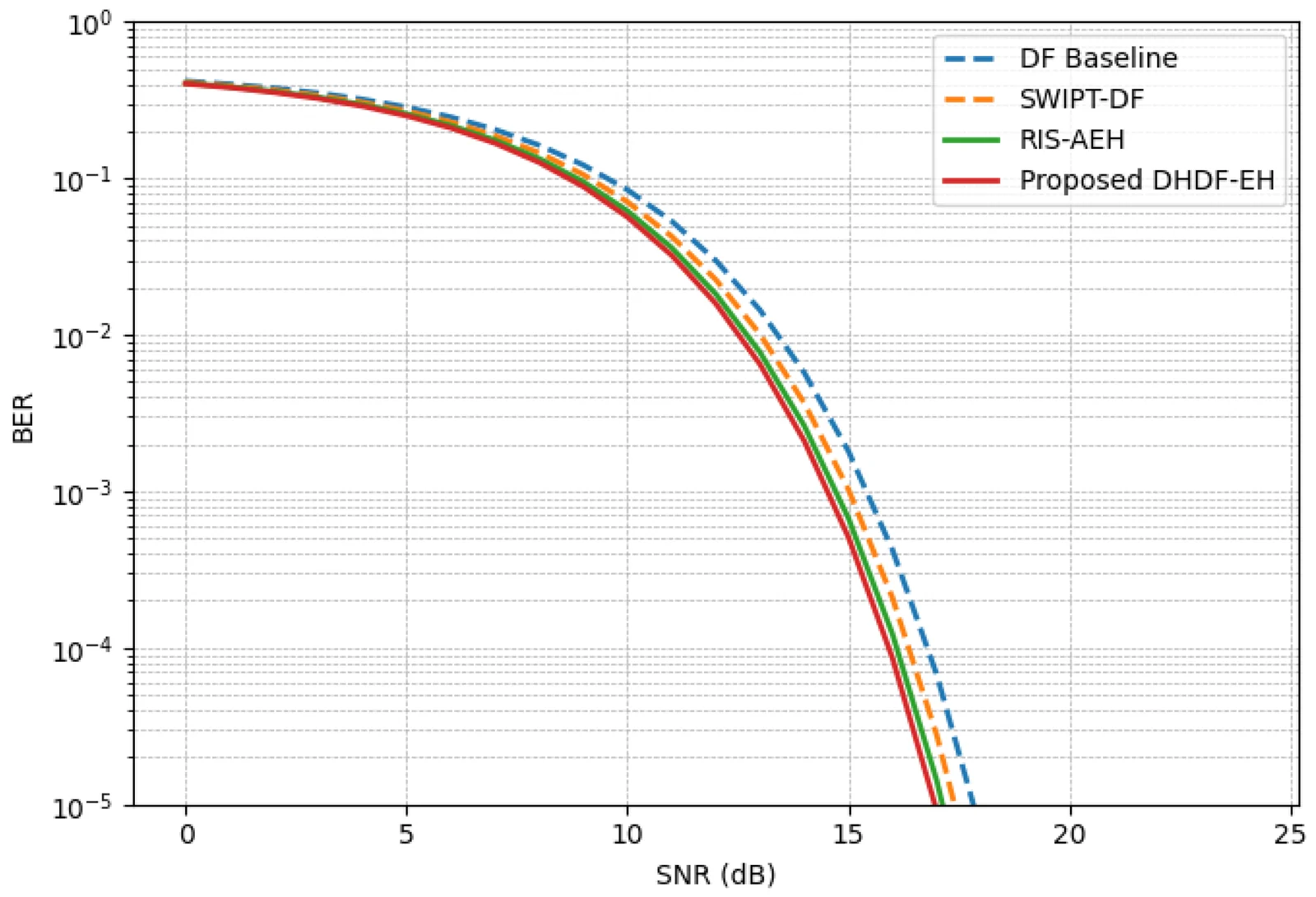
BER under α-μ Fading, when $N_{S}=N_{R}=3$, α=2, μ=2.

Figure 22 shows BER under α-μ fading scenario, where channel fluctuations are more severe and widespread; all schemes become more sensitive at lower SNR levels. The DF baseline exhibits the highest outage probability, indicating that its reliability drops under harsh conditions. By incorporating EH, SWIPT-DF improves performance by increasing the availability of the signal. RIS-AEH then further stabilizes communication through increased effective channel gain. This improves outage performance over both DF and SWIPT-DF. The real standout, however, is the proposed DHDF-EH scheme. It performs way better than the others, showing strong resilience to severe fading. This is because it keeps the lowest outage probability, thanks to its improved energy efficiency and dual-hop diversity setup.

**Figure 23:**
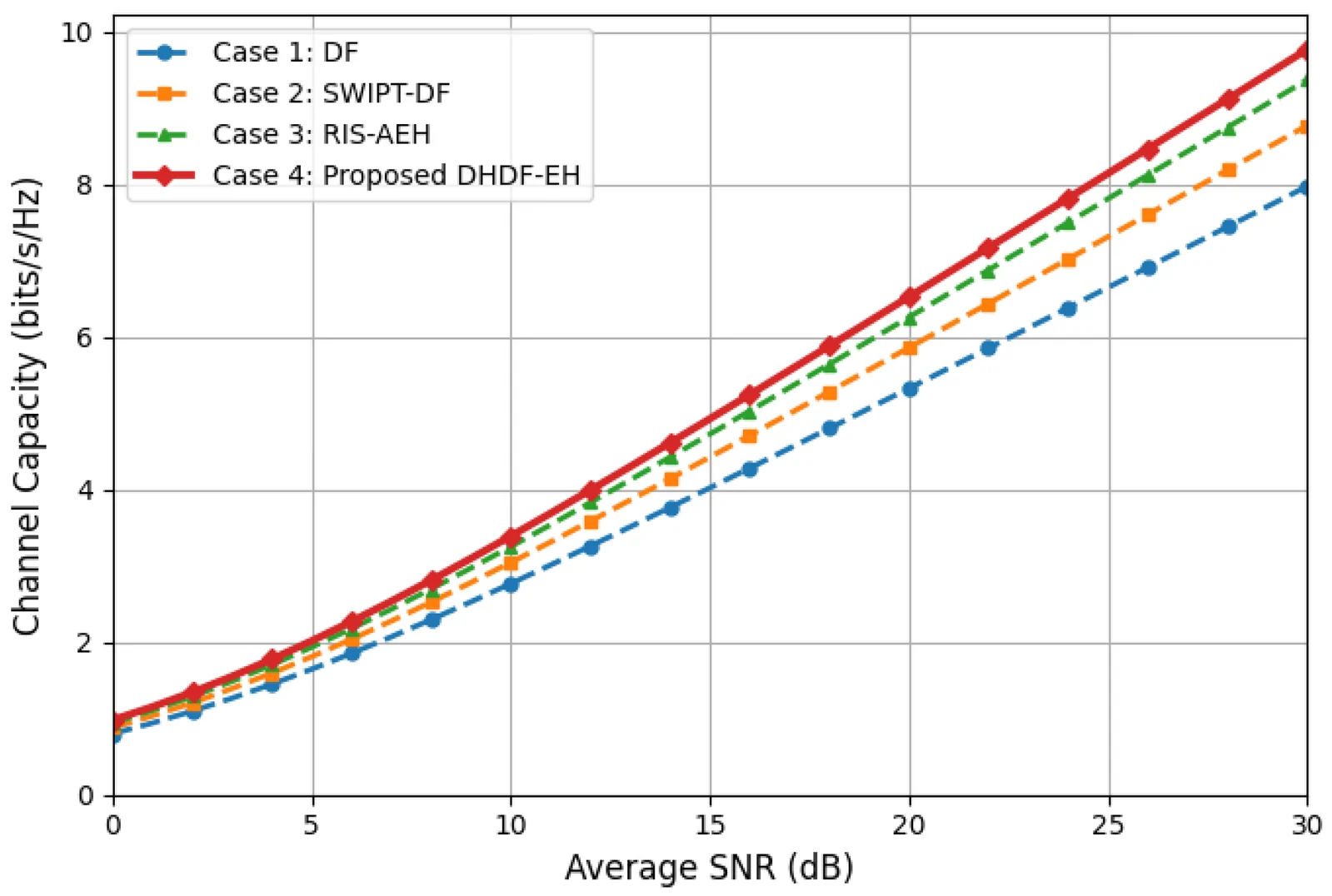
Channel Capacity under α-μ Fading, when α=2, μ=3, $N_{S}=N_{R}=3$, $P_{\mathrm{out}}=0.1$.

Figure 23 shows the performance of the proposed method with the α-μ fading channel model as a function of the average SNR. The results show that as the average SNR increases, the performance of DF improves in case 1. In case 2, the same strategy shows that SWIPT-DF also improves performance. Overall, the proposed DHDF-EH achieves the highest efficiency in improving channel capacity.

### 5.2. Probe Data

The probe data represent real-time network measurements recorded for each trial in the Monte Carlo simulation, capturing the dynamics of the 6G wireless network environment. The probe data include instantaneous SNR, received signal strength (RSS), CSI, α-μ fading parameters, EH level, relay activation status, transmission success rate, channel occupation, transmission delay, and throughput measurements. The recorded probe data are then monitored and forwarded to the Azure Kubernetes simulation framework, which dynamically controls the adaptive EH procedure, relay selection, and channel environment.

**Figure 24:**
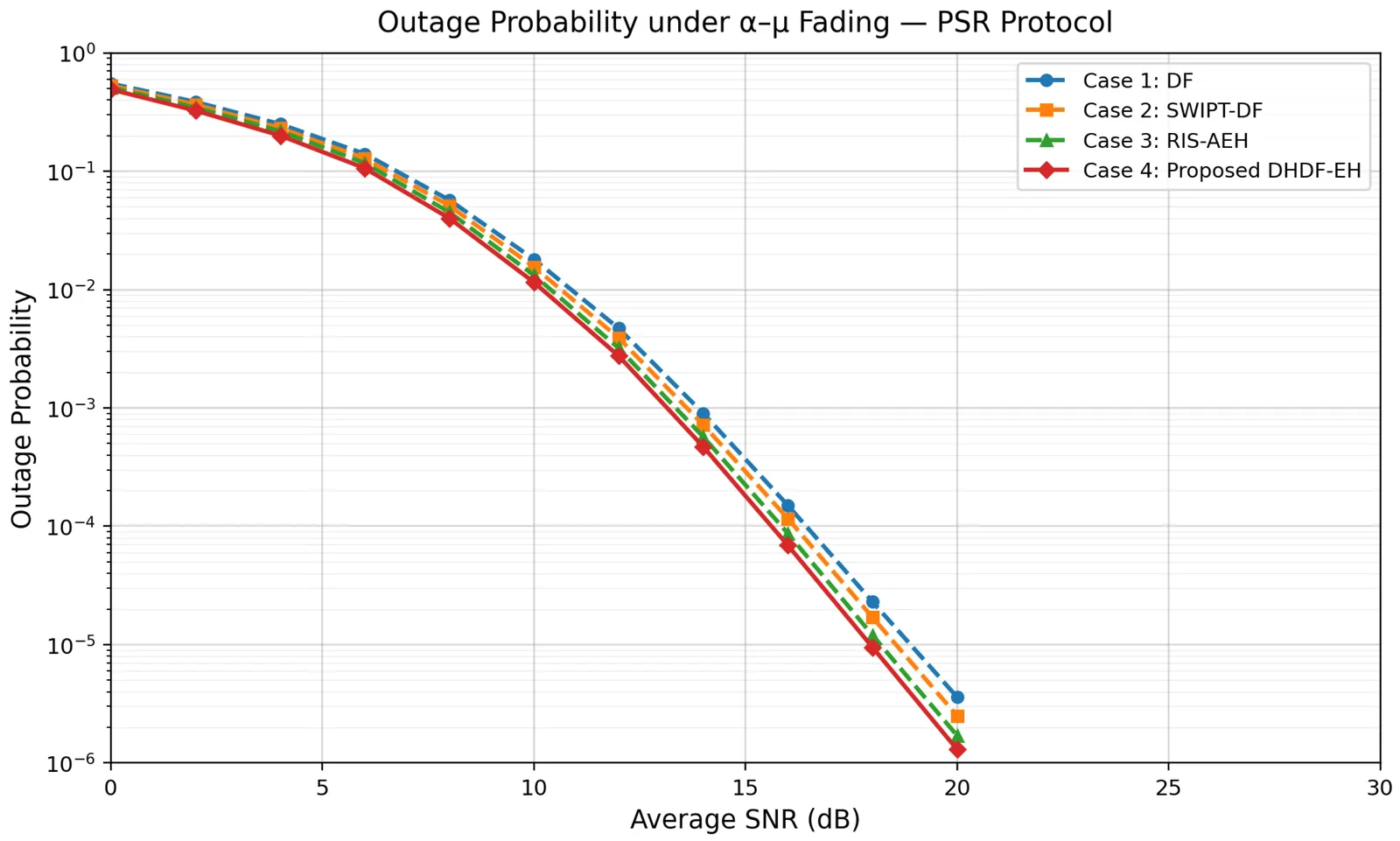
Performances of Outage Probability under α-μ Fading in Monte Carlo on AKS Signal Data with PSR Protocol, when ρ=0.5, α=3, μ=2, $N_{S}=N_{R}=3$.

Figure 24 shows the outage probability behavior of the communication systems using the PSR protocol over α-μ fading channel as the average SNR varies from 0 dB to 30 dB. We compared traditional DF, SWIPT-DF, RIS-AEH, and the proposed DHDF-EH technique. It is clear that the outage probability decreases significantly as SNR increases in all cases, since higher received signal strength improves the reliability of wireless communication. However, the proposed DHDF-EH technique achieves the lowest outage probability across all SNR values, indicating its high efficiency in enabling reliable information exchange even under extreme channel fading. This is explained by the efficient implementation of dual-hop decode-and-forward relaying and EH, which effectively uses harvested energy while maintaining stable relay operation. The RIS-AEH approach demonstrates considerable performance gain compared to the traditional DF and SWIPT-DF techniques due to the intelligent signal reflection; however, it has a higher outage probability than the proposed system. At high SNR, the outage probability of DHDF-EH becomes close to $\left(10^{-6}\right)$ while other approaches converge much more slowly.

**Figure 25:**
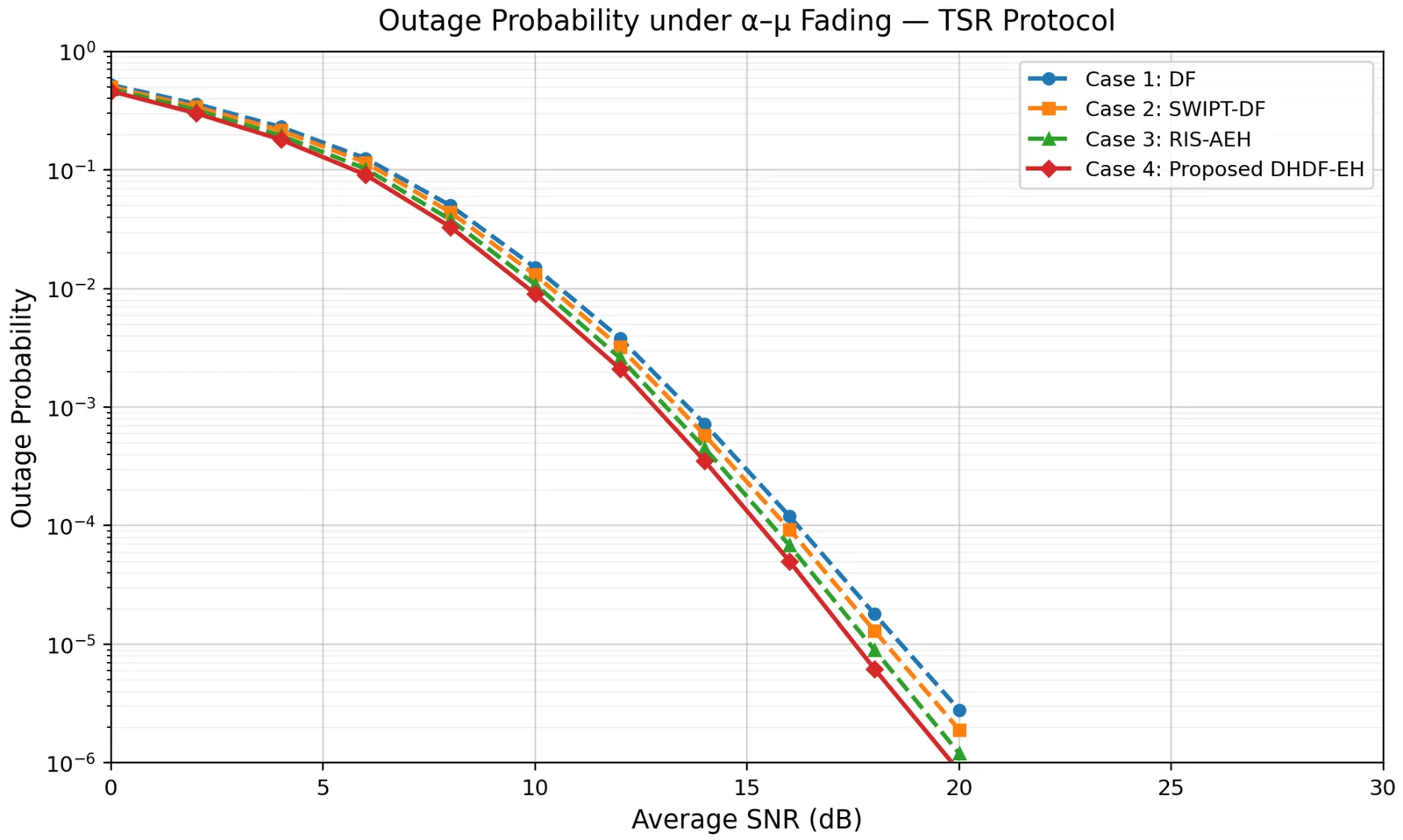
Performances of Outage Probability under α-μ Fading on Monte Carlo on AKS Signal Data with TSR Protocol, when τ=0.5, α=3, μ=2, $N_{S}=N_{R}=3$.

Figure 25 shows the outage probability behavior of the communication systems in an α-μ fading channel with TSR as the average SNR varies from 0 dB to 30 dB. We compared the traditional DF, SWIPT-DF, RIS-AEH, and the proposed DHDF-EH technique. The outage probability decreases significantly as SNR increases in all cases, since stronger received signals improve wireless communication reliability. However, the proposed DHDF-EH technique achieves the lowest outage probability across all SNR values, indicating its high efficiency in enabling reliable information exchange even under extreme channel fading. This is explained by the efficient implementation of dual-hop decode-and-forward relaying and EH, which effectively uses harvested energy while maintaining stable relay operation. The RIS-AEH approach demonstrates a considerable performance gain over the traditional DF and SWIPT-DF techniques due to intelligent signal reflection; however, it exhibits a higher outage probability than the proposed system. At high SNR, the outage probability of DHDF-EH becomes close to $\left(10^{-6}\right)$ while other approaches converge much more slowly.

## 6. Conclusions

This paper analyzed a dual-hop DF relaying RF and EH that employs adaptive transmission techniques in α-μ fading environments. We derived exact analytical expressions for outage probability, channel capacity, and bit error rate. The main advantages are improved channel capacity, reduced outage, and lower BER, as shown in the results. The key contribution is that increasing antenna diversity, transmit power, and SNR, and improving fading parameters all enhance reliability and capacity. These findings show that gains come from greater diversity and better signal quality. Applying a realistic fading model, employing efficient EH, using moment-generating functions, and adopting adaptive transmission strategies can significantly improve the performance of cooperative relay systems. We simulated all results using an analytical mathematical model in the AKS environment and showed the efficiency of the proposed methods. Furthermore, our approach provides a robust, accurate framework for evaluating DF-based energy-harvesting systems across various fading conditions. The strong alignment between simulation and analysis confirms the system’s reliability. These results support the development of efficient, adaptive wireless systems for future technologies, including 6G.

## Figures and Tables

**Table 1:** Common fading models as special cases of the α-μ model.

Fading Distributions	α	μ
Nakagami-*m*	2	*m*
Rayleigh	2	1
One-sided Gaussian	2	$\frac{1}{2}$
Weibull	$\frac{\alpha }{2}$	1
Negative exponential	1	1

**Table 2:** Experimental Simulation in Monte Carlo.

Parameter	Configuration
Simulation Environment	6G-enabled wireless communication system
Simulation Platform	AKS
Simulation Method	Monte Carlo simulation
Channel Model	Generalized α-μ fading channel
Relay Scheme	EH Decode-and-Forward (EH-DF)
Benchmark Methods	Conventional DF, SWIPT-DF, RIS-EH
Source Antennas ($N_{S}$)	3
Relay Antennas ($N_{R}$)	3
Transmit Power ($P_{s}$)	30–70 dBm
Power Splitting Ratio, Time Switching Ratio	0.5
EH Conversion Efficiency	0.75
Fading Parameters	α, μ (varied during simulations)
Monte Carlo Iterations	$N_{\mathrm{MC}}$ independent channel realizations
Execution Mode	Parallel Monte Carlo trials on AKS
Performance Metrics	Outage Probability, BER, Channel Capacity
Validation	Comparison between analytical and Monte Carlo results
Parameter Analysis	Effects of antenna configuration, transmit power, energy harvesting parameters, and fading severity

## Data Availability

The raw data supporting the conclusions of this article will be made available by the authors on request.
